# Characterisation of Modular Polyketide Synthases Designed to Make Pentaene Analogues of Amphotericin B

**DOI:** 10.3390/molecules29061396

**Published:** 2024-03-21

**Authors:** Yuhao Song, Mark Hogan, Jimmy Muldoon, Paul Evans, Patrick Caffrey

**Affiliations:** 1 School of Biomolecular and Biomedical Science, University College Dublin, D04 V1W8 Dublin, Ireland; yuhao.song@ucdconnect.ie (Y.S.); mark.hogan@ucdconnect.ie (M.H.); 2 School of Chemistry and Centre for Synthesis and Chemical Biology, University College Dublin, D04V1W8 Dublin, Ireland; jimmy.muldoon@ucd.ie (J.M.); paul.evans@ucd.ie (P.E.)

**Keywords:** glycosylated polyene macrolides, antifungal antibiotics, amphotericin B, modular polyketide synthase, synthetic biology

## Abstract

Glycosylated polyene macrolides are important antifungal agents that are produced by many actinomycete species. Development of new polyenes may deliver improved antibiotics. Here, *Streptomyces nodosus* was genetically re-programmed to synthesise pentaene analogues of the heptaene amphotericin B. These pentaenes are of interest as surrogate substrates for enzymes catalysing unusual, late-stage biosynthetic modifications. The previous deletion of amphotericin polyketide synthase modules 5 and 6 generated *S. nodosus* M57, which produces an inactive pentaene. Here, the chain-terminating thioesterase was fused to module 16 to generate strain M57-16TE, in which cycles 5, 6, 17 and 18 are eliminated from the biosynthetic pathway. Another variant of M57 was obtained by replacing modules 15, 16 and 17 with a single 15–17 hybrid module. This gave strain M57-1517, in which cycles 5, 6, 15 and 16 are deleted. M57-16TE and M57-1517 gave reduced pentaene yields. Only M57-1517 delivered its predicted full-length pentaene macrolactone in low amounts. For both mutants, the major pentaenes were intermediates released from modules 10, 11 and 12. Longer pentaene chains were unstable. The novel pentaenes were not glycosylated and were not active against *Candida albicans*. However, random mutagenesis and screening may yet deliver new antifungal producers from the M57-16TE and M57-1517 strains.

## 1. Introduction

**Figure 1:**
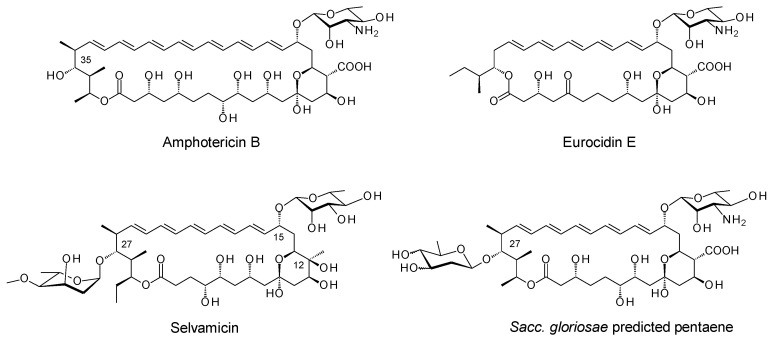
Structures of amphotericin B and pentaenes.

Amphotericin B (Figure 1) is a highly active but toxic antifungal and antiparasitic drug that is produced by *Streptomyces nodosus* [1]. There is interest in developing related glycosylated polyene macrolides as improved antibiotics, preservatives and crop protection agents. Analogues of these natural product compounds can be obtained by engineered biosynthesis, by chemical modification or by a combination of both methods.

The macrolactone core of amphotericin B is constructed by a complex polyketide synthase (PKS) composed of 18 extension modules (Figure S1 in the Supplementary Materials). The ring is oxidised by cytochrome P450s and glycosylated with mycosamine (Figure S2). These late steps are common to most of the known glycosylated polyene macrolides. In 2016, the Clardy group discovered selvamicin, a pentaene with unusual late modifications (Figure 1) [2]. Selvamicin lacks the exocyclic carboxyl group that results from the P450-catalysed oxidation of a methyl branch. Instead, the methyl-bearing methine at C12 is hydroxylated by a 2-ketoglutarate-dependent dioxygenase. With polyenes in general, the absence of an exocyclic carboxyl group reduces toxicity [3]. The impact of C12 hydroxylation on biological activity has yet to be reported. Selvamicin glycosylation is also unusual in that the macrolactone ring is modified at C15 with D-rhamnose and at C27 with 4-*O*-methyl-L-digitoxose. The replacement of the standard D-mycosamine with D-rhamnose accounts for the relatively low antifungal activity of selvamicin. 4-*O*-methyl-L-digitoxose increases water solubility. Similar glycosylation does not impair the antifungal activity of the minor nystatin congener, nystatin A3 [4]. Interestingly, bioinformatic predictions suggest that *Saccharopolyspora gloriosae* can synthesise a selvamicin-related pentaene that is modified at C27 with a 2, 6-dideoxy-D-hexose, possibly D-olivose (Figure 1) [1]. This suggests that alternative sugars might be added at this position by enzymatic glycosylation, at least with some polyenes. The well-characterised pentaene eurocidin (Figure 1) is not amenable to this modification.

**Figure 2:**
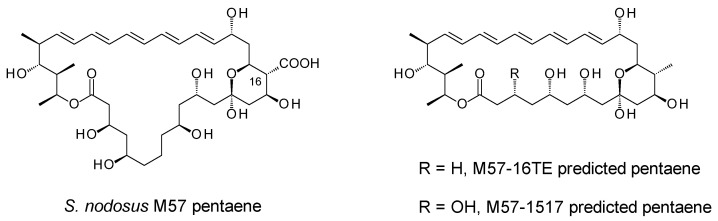
Structures of pentaene macrolactones from engineered *S. nodosus* strains.

We attempted to produce new antifungals by expressing enzymes catalysing unusual polyene late modifications in the amphotericin producer, *Streptomyces nodosus*. While polyene extending glycosyltransferases (GTs) have been used to add a second sugar to mycosamine, the enzymatic glycosylation of the C35 position of amphotericin B has remained elusive [5]. To investigate this step further, we set out to construct *S. nodosus* mutants that synthesise macrolactones analogous to the aglycones of selvamicin and the *Sacc. gloriosae* pentaene. A pentaene analogue of amphotericin B was previously obtained by engineering the *amphC* PKS gene in *S. nodosus* [6]. This gave strain *S. nodosus* M57, in which extension cycles 5 and 6 were deleted from the polyketide biosynthetic pathway (Figure S3). Strain M57 produced high yields of an inactive pentaene analogue, in which the polyene chain was shorter than the polyol chain (Figure 2). In this pentaene, the methyl branch at C16 was oxidised to a carboxyl group, but less than 10% of the macrolactone was glycosylated with mycosamine [7]. The current work aimed to engineer strain M57 further, by deleting two of the last four modules to reduce the length of the polyol chain by four carbon atoms. This would give new macrolactones. One approach aimed to fuse the chain-terminating thioesterase (TE) to module 16, thereby eliminating extension cycles 17 and 18. A second approach set out to replace modules 15, 16 and 17 with a single 15/17 hybrid module. Both PKSs were expected to give ring-contracted pentaene macrolactones similar in structure to that of selvamicin (Figure 2). The two *S. nodosus* strains could then be assessed as hosts for studies in combinatorial biosynthesis.

## 2. Results

### 2.1. Fusion of TE to Module 16

**Figure 3:**
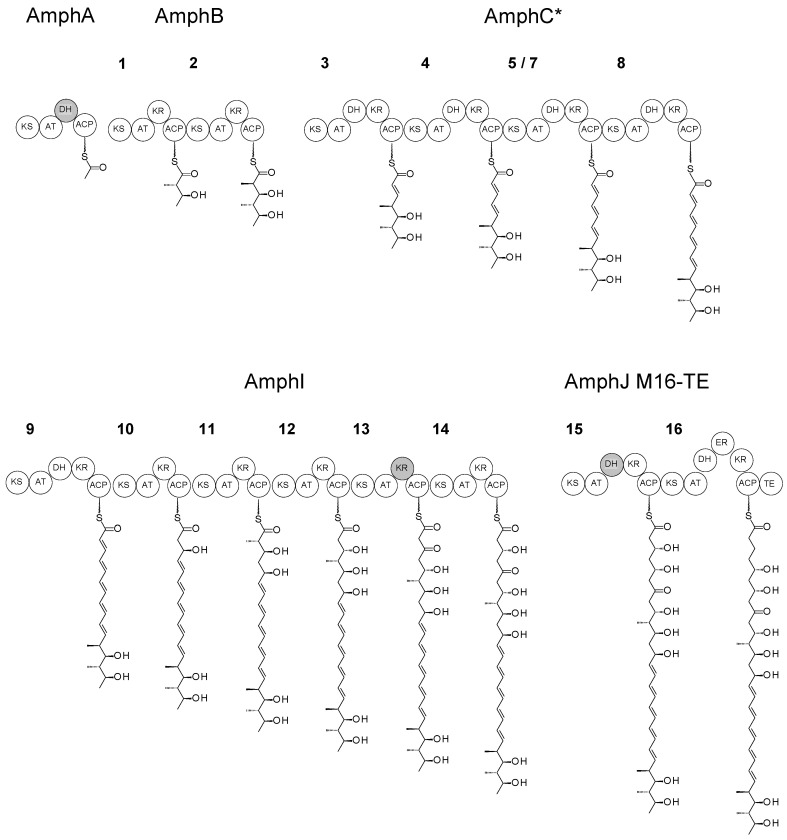
Modular structure of the PKS and intermediates in *S. nodosus* M57-16TE. The tetramodular AmphC* protein replaces the native hexamodular AmphC, and the AmphJ-M16TE protein replaces the trimodular AmphJ. Inactive domains are shaded grey. Note that 5/7 is a hybrid module (KS5-AT7-DH7-KR7-ACP7) that replaces modules 5, 6 and 7 of the full-length PKS assembly line. Numbering of other modules is as for the native amphotericin PKS (Figure S1).

Module 16 of the amphotericin PKS contains a full complement of domains, ketosynthase (KS), acyltransferase (AT), dehydratase (DH), enoylreductase (ER), ketoreductase (KR) and acyl carrier protein (ACP). The coding sequences for the ER16-KR16-ACP16 domains and the TE domain were separately amplified by PCR and cloned into pUC118 (Figure S4). The ACP16-TE fusion point aligns closely with the junction used in a productive ACP13-TE hybrid derived from the stambomycin PKS [8] (Figure S5). The DNA fragment encoding ER16-KR16-ACP16-TE was assembled and cloned into phage KC-UCD1 to make phage KC16-TE. This integrated into the chromosome of *S. nodosus* M57 to give *S. nodosus* M57-16TE (Figure S6). PCR analysis revealed that this recombinant contained the required change in the *amphJ* PKS gene (Figure S7). This gives the PKS shown in Figure 3, where the chain-terminating TE is fused to module 16.

### 2.2. Deletion of Two Modules from the AmphJ PKS Protein

A second approach aimed to replace modules 15, 16 and 17 with a single hybrid module. The coding sequences for KS15 and AT17-DH^0^17-KR17-ACP17 were amplified by PCR and used to assemble a gene for AmphJ*, a single-module PKS protein. This gene was initially cloned into a pIAGO expression vector to create pIAGO-M1517 (Figure S8). The KS15-AT17 junction was the same as that used to fuse KS5 and AT7 in *S. nodosus* M57, a strain that produces high levels of pentaenes (Figure S9). The pIAGO-M1517 plasmid was transformed into *S. nodosus* M57. The transformant produced the M57 pentaenes and the predicted M57-1517 pentaene, presumably because both AmphJ* and AmphJ were active. However, the pentaene mixture was complex, so a clean exchange of AmphJ* for AmphJ was carried out by chromosomal gene replacement.

**Figure 4:**
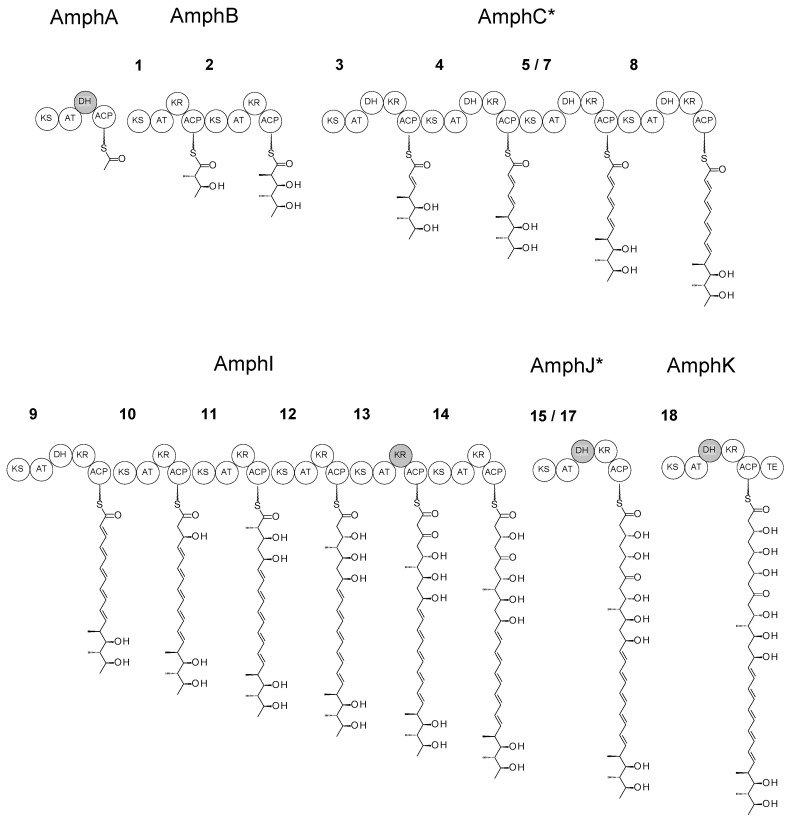
Modular structure of the PKS and intermediates in *S. nodosus* M57-1517. The tetramodular AmphC* protein replaces the native hexamodular AmphC and the unimodular AmphJ* protein replaces the trimodular AmphJ. Inactive domains are shaded grey. Note that 5/7 is a hybrid module (KS5-AT7-DH7-KR7-ACP7) that replaces modules 5, 6 and 7 of the full-length PKS assembly line. 15/17 is a hybrid module (KS15-AT17-DH17-KR17-ACP17) that replaces modules 15, 16 and 17. Numbering of other modules is as for the native amphotericin PKS (Figure S1).

The KS15-AT17 coding region was cloned from pIAGO-M1517 into KC-UCD1, and the recombinant phage was used to replace the trimodular AmphJ protein gene with a unimodular AmphJ* gene (Figure S10). Replacement in *S. nodosus* M57 gave *S. nodosus* M57-1517. The structure of the engineered PKS is shown in Figure 4.

### 2.3. Analysis of Pentaenes from S. nodosus M57-16TE and S. nodosus M57-1517

**Figure 5:**
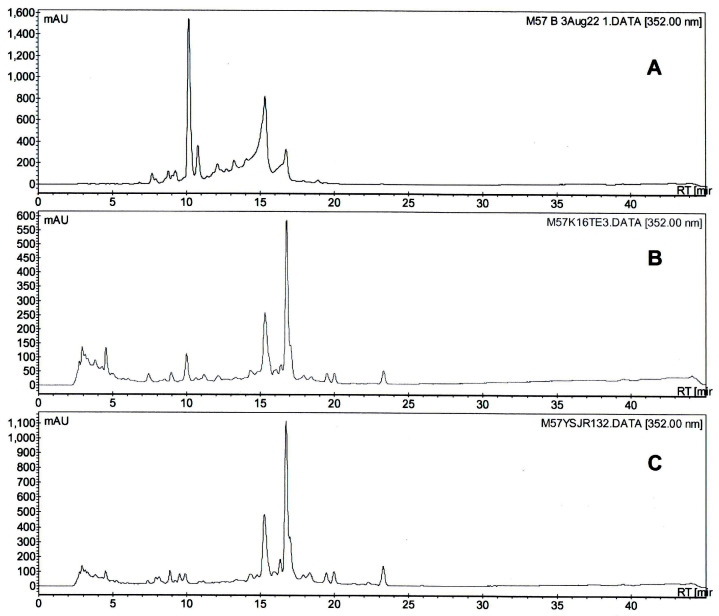
HPLC analysis of pentaenes from (**A**) *S. nodosus* M57, (**B**) *S. nodosus* M57-16TE, (**C**) *S. nodosus* M57-1517.

Pentaenes from *S. nodosus* M57-16TE and *S. nodosus* M57-1517 were partially purified by solid-phase extraction and analysed by HPLC (Figure 5). Both mutants produced two major pentaenes. There was a 20-fold decrease in yield relative to the total pentaene production by the *S. nodosus* M57 parent strain. The M57-16TE and M57-1517 pentaenes showed no antifungal activity in bioassays against *Candida albicans*.

The new pentaenes were unstable and decomposed when partially purified material in methanol containing 0.1% (*v*/*v*) formic acid was concentrated in a vacuum centrifuge at temperatures of 40 °C. The main pentaenes converted to forms that retained the pentaene chromophore but had later HPLC retention times. For both M57-16TE and M57-1517, the polar pentaene peaks with retention times of 15 min (Figure 5) disappeared very quickly, whereas the more abundant pentaenes, with retention times of 17 min, were stable. Bacillaenes, linear hexaenes and pentaenes synthesised by *Bacillus subtilis* are notoriously unstable [9,10].

The pentaenes from *S. nodosus* M57-16TE were partially purified by semi-preparative HPLC. LC-MS analysis revealed that the more polar leading peak contained a series of ions with *m*/*z* values of 603.1, 621.1, 639.1, 657.1 and 675.1 (Figure S11B). The calculated molecular mass of the predicted macrolactone product was 592.3611 (C_33_H_52_O_9_). The observed molecular masses may have resulted from the auto-oxidation of the unstable pentaene species [11]. Attempts to purify this molecule for NMR spectroscopic analysis were unsuccessful. The less polar trailing pentaene peak from M57-16TE gave ions consistent with the products of modules 10, 11 and 12 (Figure S11C; see below).

The new pentaenes from M57-1517 were partially purified by solid-phase extraction. Preliminary LC-MS analysis revealed that the M57-1517 extract contained an ion consistent with the expected macrolactone ([M + Na]^+^ = 631.3449; calculated for C_33_H_52_O_10_Na = 631.3458). There was no evidence for pentaene macrolactones containing exocyclic carboxyl groups or mycosamine sugar residues. Further purification was carried out to isolate pentaenes from this M57-1517 mutant.

### 2.4. Analysis of Major Pentaene from S. nodosus M57-1517

**Figure 6:**
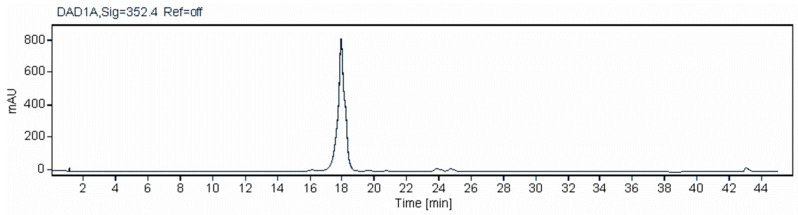
HPLC analysis of highly purified pentaene from *S. nodosus* M57-1517. A plot of A_352_ against time is shown. The diode array display of this chromatogram indicated that the pentaene was free of contaminants that absorb in the UV-visible region (Figure S12).

A new purification procedure was developed to avoid heating in 0.1% formic acid. The M57-1517 pentaenes were extracted and purified by solid-phase extraction and semi-preparative HPLC. To remove formic acid, water was added to the peak fractions to dilute the methanol concentration to 10% methanol. The pentaene was bound to a C18 solid-phase extraction cartridge and eluted with methanol. Further purification was achieved by a second semi-preparative HPLC run. Formic acid was finally removed by gel filtration in methanol on a Sephadex LH20 column. The minor pentaene was lost during the purification process. The major pentaene was more stable and was successfully purified (Figure 6). The diode array display revealed the absence of other contaminants that absorb in the UV–visible region (Figure S12).

**Figure 7:**
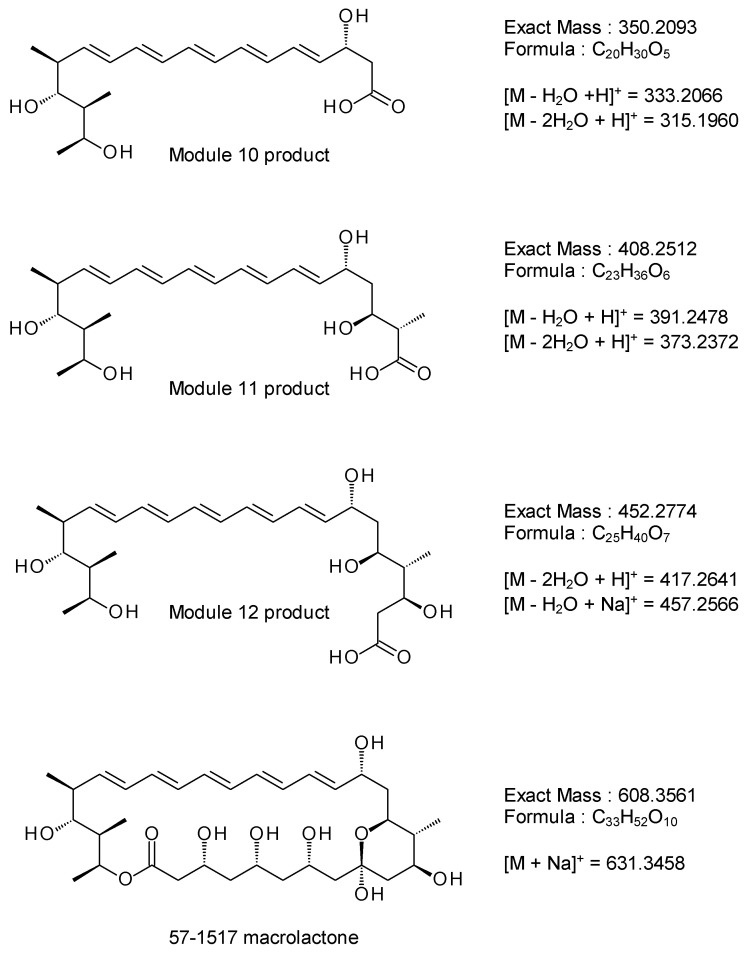
Structures of pentaene polyketide intermediates and full-length 57-1517 macrolactone. The products of extension modules 10, 11 and 12 (see Figure 3) are shown as free acids. The calculated molecular masses can be fitted to ions detected in the purified pentaene sample (see Figure S15).

LC-MS analysis of the purified pentaene revealed that the full-length macrolactone remained only as a minor component (Figures S13 and S14). The most abundant ion had an exact mass appropriate for the pentaene polyketide product of module 10 ([M − H_2_O + H]^+^ = 333.2066, [M − 2H_2_O + H]^+^ = 315.1960) (Figure 7 and Figure S15). Less abundant ions corresponded to the products of module 11 ([M − H_2_O + H]^+^ = 391.2478, [M − 2H_2_O + H]^+^ = 373.2372) and module 12 ([M − H_2_O + Na]^+^ = 457.2566, [M − 2H_2_O + H]^+^ = 417.2641). The early release of these intermediates could occur if chain completion were to be delayed by inefficient action of the hybrid AmphJ* module or slow off-loading/cyclisation by the TE domain. The ions with *m*/*z* values of 353.2303 and 427.2674 could not be explained and may result from a non-UV active lipid contaminant.

### 2.5. Analysis of M57-1517 Pentaene by NMR Spectroscopy

The purified pentaene was dissolved in D_3_COD and characterised by 1D proton NMR spectroscopy, heteronuclear single quantum coherence (HSQC) spectroscopy, and 1H-1H-COSY spectroscopy (Figures S16–S22). Signals for all of the non-exchangeable protons in the module 10 intermediate could be identified and assigned. On the basis of the ion spectrum in Figure S15, this is the most abundant pentaene in the sample. However, the NMR spectra also provided additional evidence for contamination with a fatty acid impurity.

### 2.6. Fusion of TE Domain to Module 16 of a Heptaene-Producing Amphotericin PKS

**Figure 8:**
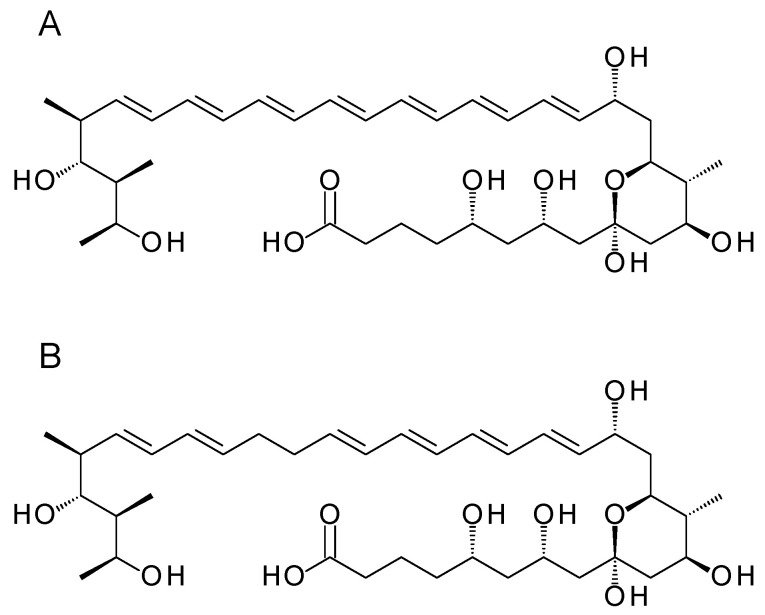
Polyenes predicted from *S. nodosus* NM-16TE. (**A**) heptaene; (**B**) tetraene.

Phage KC16-TE was used to fuse the chain-terminating TE to module 16 in a full-length heptaene/tetraene-producing amphotericin PKS. The phage integrated its genome into the chromosome of *S. nodosus* NM to give several recombinants by the same cross-over pathway as that shown in Figure S6. The resulting PKS was predicted to synthesise a heptaene and a tetraene that could not be readily cyclised (Figure 8). If the TE were to catalyse the efficient release of these linear chains by hydrolysis, the two products should be obtained in moderately high yields. However, multiple heptaenes and tetraenes were obtained in trace amounts (Figure S23). This suggests that the TE does not act on the truncated polyol chain. The series of trace polyene peaks in the HPLC chromatograms likely represent backlogs of polyketide intermediates released from late modules by a discrete editing TE or by spontaneous hydrolysis. These results indicate that the chain-terminating TE does not catalyse the efficient hydrolytic release of linear polyketide chains with a shortened polyol chain. A lack of activity towards unnatural substrates may also account for the low yields of the ring-contracted pentaene macrolactones (Figure S24).

### 2.7. Attempted Glycosylation Engineering of Pentaenes in S. nodosus M57-1517

*S. nodosus* M57-1517 retains all of the late genes involved in post-polyketide modifications, yet the full-length pentaene macrolactone was not oxidised by the AmphN P450 or modified with mycosamine by the AmphDI glycosyltransferase. The lack of glycosylation is consistent with the absence of antifungal activity. While the full-length pentaene macrolactone is a minor product of *S. nodosus* M57-1517, the more abundant linear products have the hydroxyl groups on carbon atoms corresponding to C15 and C27 in selvamicin aglycones. It was decided to investigate whether pentaene-specific mycosaminyltransferases can modify “C15” and whether SelSV glycosyltransferase can modify “C27”. The DesVII/DesVIII glycosyltransferase acts on linear analogues of the methymycin aglycone [12,13].

We previously found that heptaene amphoteronolide aglycones were modified with mycosamine by the EurDI glycosyltransferase from the pentaene producer *Streptomyces eurocidicus* and by the GloDI mycosaminyltransferase from the cryptic pentaene gene cluster in *Sacc. gloriosae* [5]. Here, we transformed *S. nodosus* M57-1517 with the expression plasmids for these pentaene glycosyltransferases, pIAGO-EurDI-DI-N-M and pIAGO-GloDI. However, no antifungal activity was detected and no new pentaenes were detected by HPLC.

*S. nodosus* M57-1517 was also transformed with the expression plasmid for the SelSV GT (pIJ-SelSV) [5] and the Salas plasmid pFL942 that contains genes for the biosynthesis of dTDP-β-L-mycarose and lower levels of dTDP-β-L-digitoxose [14]. The transformant did not produce detectable pentaenes that might represent mycarosylated or digitoxosylated forms.

In future work, it may be possible to increase the production of pentaene aglycone substrates so that low-level glycosylation can be detected and optimised.

## 3. Discussion

Many polyene macrolides have been characterised over the last 50 years [15]. Biosynthetic gene clusters for a few of these occur frequently in actinomycete genomes [1]. These are tetraenes related to pimaricin, heptaenes and degenerate heptaenes related to amphotericin B and nystatin, and aromatic heptaenes related to candicidin and partricin. Pentaenes are a relatively small group. Unglycosylated methylpentaenes related to filipin are common, but these target cholesterol-containing eukaryotic membranes in general [16]. Until recently, eurocidin (Figure 1) was the only example of a highly active glycosylated pentaene with specificity for ergosterol-containing fungal membranes [17]. Eurocidin does not have a hydroxyl group corresponding to C27 of selvamicin (or C35 of nystatin) that could be glycosylated with L-digitoxose. Pimaricin and the aromatic heptaenes are also unsuitable as surrogate substrates as they lack this acceptor hydroxyl.

This study set out to develop an *S. nodosus* strain as a chassis host organism for investigating unusual late biosynthetic modifications of naturally occurring pentaenes. We designed two different versions of the amphotericin PKS to synthesise ring-contracted pentaene macrolactones. Both gave low levels of novel compounds. Similar reductions in yield have been observed with other systems [8]. In deleting modules from amphotericin PKS proteins, care was taken to use junctions that were considered optimal. The likely problem was the TE domain, which has been considered a bottleneck in other engineered PKSs [18].

Polyene chain-terminating TEs have been studied intensively [18,19]. These domains belong to a distinct phylogenetic group. They are closely related to each other but not to the erythromycin and pikromycin PKS TE domains. The structure of the pimaricin TE domain has been determined [18]. This indicates that polyene PKS TE domains are adapted to act on their native substrates and may have a lower tolerance towards altered polyketide chains. A single amino acid substitution can affect activity; for example, the L170R mutant of the pimaricin TE gave linear rather than cyclised products. Jiang et al. suggest that polyene PKS TE domains catalyse the formation of the hemiketal ring as well as the macrocyclisation [19]. Our work suggests that the native amphotericin TE can tolerate shortening of the polyene unit but not the polyol unit.

Both M57-16TE and M57-1517 accumulated polyketide intermediates, specifically the products of modules 10, 11 and 12. These may be more stable than later intermediates or more easily released by a discrete editing thioesterase.

LC-MS provided evidence that the M57-1517 PKS produces low levels of the cyclised ring-contracted pentaene macrolactone. This was apparently not recognised or modified by AmphN P450, AmphDI mycosaminyltransferase or AmphL P450 enzymes (see Figure S2). The yields of this pentaene were so low that it was not possible to detect glycosylation by pentaene glycosyltransferases such as GloDI and EurDI mycosaminyltransferases or by the SelSV glycosyltransferase that adds methyl-L-digitoxose to C27 of the selvamicin macrolactone. Current knowledge of structure–activity relationships in polyenes suggests that a mycosaminylated form of the M57-1517 pentaene should have antifungal activity. Improvements in the yield of this pentaene might be obtained by engineering of the TE domain. Alternatively, the M16-TE and M57-16TE strains may be useful for accelerated evolution studies [20]. Productive strains might be obtained by random mutagenesis followed by screening for the restoration of antifungal activity.

## 4. Materials and Methods

### 4.1. DNA Methods

PCR was carried out using Phusion DNA polymerase (Waltham, MA, USA). Amplified DNA was purified using a Qiagen QiaEx kit (Finlo, The Netherlands). Restriction enzyme digestions and ligations were carried out according to the manufacturers’ instructions. Transformations were carried out by electroporation or by the CaCl_2_ method with *E. coli* DH5α as a general cloning host. Plasmids were purified using Qiagen or GeneJet mini-prep kits (GeneJet, Taoyuan, Taiwan). Resequencing was carried out by Source Bioscience. Phage KC-UCD1-mediated gene replacements were carried out as described previously [21,22].

### 4.2. Extraction of Polyenes

For polyene production, a starter culture of the *S. nodosus* strain was grown on tryptic soy (TS) medium at 30 °C with shaking for 40 h. This culture was used to inoculate 250 mL flasks containing 100 mL volumes of the production medium, fructose–dextrin–soya flour medium, containing 5% (*w*/*v*) Amberlite XAD16 [7]. Flasks also contained stainless steel springs to disperse mycelium and increase aeration. The production cultures were shaken at 30 °C for six days. Then, mycelial cells and Amberlite XAD16 resin beads were sedimented by centrifugation. The resulting pellets were extracted twice for 2 to 3 h with methanol. The volume of methanol used for each extraction was equivalent to the original culture volume. The combined methanol extracts were concentrated by rotary evaporation. When most of the methanol evaporated off, pentaenes remained suspended in the residual water. Pentaenes were applied to a 12 mL Supelco Discovery DSC-18 solid-phase extraction (SPE) column equilibrated with 10% (*v*/*v*) methanol in H_2_O. Sample components were sequentially eluted using 50%, 60%, 80% and 100% methanol solutions. Pentaene was collected in the 50% and 60% methanol elutions. Pentaenes were further purified by semi-preparative HPLC and by gel filtration using a Sephadex LH20 column (2.5 × 45 cm) (Sephadex, Los Angeles, CA, USA) equilibrated with methanol. The flow rate was 1 mL min^−1^.

### 4.3. High Performance Liquid Chromatography (HPLC)

HPLC was used for the analysis of polyenes and for purification on a semi-preparative scale. The Varian ProStar and Agilent analytical HPLC systems (Agilent, Santa Clara, CA, USA) were used with an Agilent Zorbax SB-C18 column (9.4 × 150 mm, 5 mm). Solvent A was 0.1% (*v*/*v*) formic acid in H_2_O and solvent B was 0.1% (*v*/*v*) formic acid in methanol. A flow rate of 4 mL min^−1^ was used. The column was equilibrated with 50% B. The 500 μL sample was loaded and components were separated by applying a gradient of 60 to 90% B over 35 min. Pentaenes were identified by the characteristic UV–visible absorption spectrum revealed by the diode array display detector. For preparative chromatography, pentaene peaks were collected as 4 mL fractions. For analytical HPLC, the same method was used with 100 μL sample loading.

### 4.4. Mass Spectrometry

Mass spectrometry was carried out using an Agilent 6546 series Q-TOF LC/MS system equipped with an Agilent Jetstream Electrospray ionisation source, coupled to an Agilent 1260 Infinity prime II LC system.

### 4.5. NMR Spectroscopy

Approximately 2 mg of the purified sample was dissolved in 0.65 mL of deuterated methanol (CD_3_OD) and filtered. One-dimensional (^1^H), and two-dimensional (^1^H-^1^H-COSY, ^1^H-^13^C-HSQC and TOCSY) spectra were collected using an Agilent DD2 500 MHz console NMR spectrometer equipped with a OneNMR probe. Spectra were recorded at 25 °C. Analyses were carried out by Dr Yannick Ortin, UCD School of Chemistry.

### 4.6. Tests for Antifungal Activity

*Candida albicans* ATCC10231 was grown overnight at 30 °C 180 rpm on yeast medium. A 0.5 mL volume of this overnight culture was added to 100 mL of molten cooled (~50 °C) yeast medium agar (3 g of malt extract, 3 g of yeast extract, 5 g of peptone, 10 g of glucose and 20 g of agar per litre) and mixed. The agar was poured into sterile Petri dishes and allowed to set. Wells were punched in the agar using a sterile cork borer. Test samples were pipetted into the wells and the plate was incubated base down in a 30 °C oven incubator. A lawn of *Candida albicans* appeared after overnight incubation. Wells containing samples with antifungal activity gave a clear inhibition zone.

## 5. Conclusions

The reprogramming of the amphotericin PKS gave pentaene aglycones that are analogous to the macrolactone cores of selvamicin and a predicted *Sacc. gloriosae* pentaene. These new compounds will be useful for investigating polyene glycosylation engineering. Further work will be required to increase yields and prevent the accumulation of incomplete polyketide intermediates.

## Data Availability

All data are available in the article and the supporting information.

## Supplementary Materials

The following supporting information can be downloaded at: https://www.mdpi.com/article/10.3390/molecules29061396/s1, Figure S1: Amphotericin PKS; Figure S2: Late stages in amphotericin biosynthesis; Figure S3: *S. nodosus* M57 pentaene PKS; Figure S4: Fusion of ER16-KR16-ACP16 and TE coding sequences; Figure S5: Fusion point between ACP16 and the chain-terminating TE in *S. nodosus* M57-16TE; Figure S6: Integration of KC-M16TE into *S. nodosus* M57 chromosome; Figure S7: PCR analysis of *S. nodosus* M57-16TE; Figure S8: Construction of a coding sequence for a single-module AmphJ* protein; Figure S9: Fusion point between KS15 and AT17 in *S. nodosus* M57-1517; Figure S10: Replacement of gene for trimodular AmphJ protein with gene for single-module AmphJ* protein; Figure S11: LC-MS analysis of pentaenes from *S. nodosus* M57-16TE; Figure S12: HPLC analysis of highly purified pentaene from M57-1517; Figure S13: LC-MS analysis of HPLC-purified pentaene from M57-1517; Figure S14: Detection of full-length pentaene macrolactone from M57-1517; Figure S15: Positive ion mass spectrum of the major A_352_ pentaene peak from *S. nodosus* M57-517; Figure S16: Ring numbering for full-length pentaene macrolactone from *S. nodosus* M57-1517; Figure S17: Proton NMR spectrum of highly purified pentaene from *S. nodosus* M57-1517; Figure S18: Expansion of the 6.6 to 4.7 ppm region of the proton NMR spectrum of the purified pentaene; Figure S19: ^1^H-^13^C-HSQC NMR spectrum (0.2–6.5 ppm/10–140 ppm region) for pentaene (500 MHz, CD_3_OD, 25 °C); Figure S20: ^1^H-^1^H-gCOSY NMR spectrum for the highly purified pentaene (500 MHz, CD_3_OD, 25 °C); Figure S21: Expansion of the 4.7 to 3.1 ppm region of the proton NMR spectrum of the purified pentaene; Figure S22: Expansion of the 2.7 to 0.8 ppm region of the proton NMR spectrum of the purified pentaene; Figure S23: HPLC analysis of polyenes from *S. nodosus* NM-16TE; Figure S24: Schematic diagram showing action of amphotericin PKS TE against various substrates; Table S1: Sequences of oligonucleotide primers.
