# Supplementary material for: Characterisation of Modular Polyketide Synthases Designed to Make Pentaene Analogues of Amphotericin B
Source: Molecules. 2024 Mar 21;29(6):1396. doi: 10.3390/molecules29061396 (PMC10974182; doi:10.3390/molecules29061396)
Supplement: Supplementary file 1 [file molecules-29-01396-s001.zip › molecules-2918286-supplementary.pdf]

Supplementary Information

Characterization of modular polyketide synthases designed to make pentaene analogues of amphotericin B

Yuhao Song [1], Mark Hogan[1], Jimmy Muldoon [2], Paul Evans [2], and Patrick Caffrey [1,*]

[1]School of Biomolecular and Biomedical Science, University College Dublin, D04 V1W8, Ireland; yuhao.song@ucdconnect.ie (Y. S.); mark.hogan@ucdconnect.ie (M. H.)

[2]School of Chemistry and Centre for Synthesis and Chemical Biology, University College Dublin, D04V1W8 Dublin, Ireland; jimmy.muldoon@ucd.ie (J. M.); paul.evans@ucd.ie (P. E.)

*Correspondence: patrick.caffrey@ucd.ie (P. C.); Tel.: +353-1-716-2618

Index

Table S1. Sequences of oligonucleotide primers

Fig. S1 Amphotericin PKS
Fig. S2 Late stages in amphotericin biosynthesis
Fig. S3 *S. nodosus* M57 pentaene PKS
Fig. S4 Fusion of ER16-KR16-ACP16 and TE coding sequences
Fig. S5 Fusion point between ACP16 and the chain-terminating TE in *S. nodosus* M57-16TE.
Fig. S6 Integration of KC-M16TE into *S. nodosus* M57 chromosome
Fig. S7 PCR analysis of *S. nodosus* M57-16TE.
Fig. S8 Construction of a coding sequence for a single-module AmphJ* protein.
Fig. S9 Fusion point between KS15 and AT17 in *S. nodosus* M57-1517.
Fig. S10 Replacement of gene for trimodular AmphJ protein with gene for single-module AmphJ* protein.
Fig. S11 LC-MS analysis of pentaenes from *S. nodosus* M57-16TE
Fig. S12 HPLC analysis of highly purified pentaene from M57-1517.
Fig. S13 LC-MS analysis of HPLC-purified pentaene from M57-1517.
Fig. S14 Detection of full-length pentaene macrolactones from M57-1517
Fig. S15 Positive ion mass spectrum of the major A_{352} pentaene peak from *S. nodosus* M57-517.
Fig. S16 Ring numbering for full-length pentaene macrolactone from *S. nodosus* M57-1517.
Fig. S17. Proton NMR spectrum of highly purified pentaene from *S. nodosus* M57-1517.
Fig. S18 Expansion of the 6.6 to 4.7 ppm region of the proton NMR spectrum of the purified pentaene.
Fig. S19 ^{1}H-^{13}C-HSQC NMR spectrum (0.2-6.5 ppm/10-140 ppm region) for pentaene (500 MHz, CD_3OD, 25 °C).
Fig. S20 ^{1}H-^{1}H-gCOSY NMR spectrum for the highly purified pentaene (500 MHz, CD_3OD, 25 °C).
Fig. S21 Expansion of the 4.7 to 3.1 ppm region of the proton NMR spectrum of the purified pentaene.
Fig. S22 Expansion of the 2.7 to 0.8 ppm region of the proton NMR spectrum of the purified pentaene.
Fig. S23 HPLC analysis of polyenes from *S. nodosus* NM-16TE.
Fig. S24 Schematic diagram showing action of amphotericin PKS TE against various substrates.

Oligonucleotide	Sequence 5′ to 3′
MHF1	GATCAAGCTTCTGCAGTCAACTTCCGCGATGTCCTCAAC
MHR1	AGATCTGAGCTCGTCCAGGAGGTGCTCGGCGAGCTG
MHF2	AACGAGGA**G**CT**C**GGCGACCTGTCCGCCTCCGGCC
MHR2	TCGAGAATTCGGATCCCTGCTACTCCTTGGGAGAGTGGTGA
M16Chk	GAGGTGCGCATCGGTATCCGTG
YJF2	GTTCTCTAGACTGCAGAGGAGAGCGCGAAGTGATGGAGCAGAC
JK15R	GAAGGAGCTCACGCCGGAGCGGCGGGGGCG
JK17F	CGGTGTGAGCTCCTTCGGCATCAGCGGCACCAAC
YJR	GTACAAGCTTCCTCAGATGTTTCGCACGTAG
I-F	GAGCGGTACCAGAAGGTGTTCC
I-R	CTTCGCGCTACAGGTCGGACTG
K-F	CACCCCAACTACGTGCGAAACATC
K-R	GGCCAGGACCATCTTGATGAC

Table S1. Sequences of oligonucleotide primers

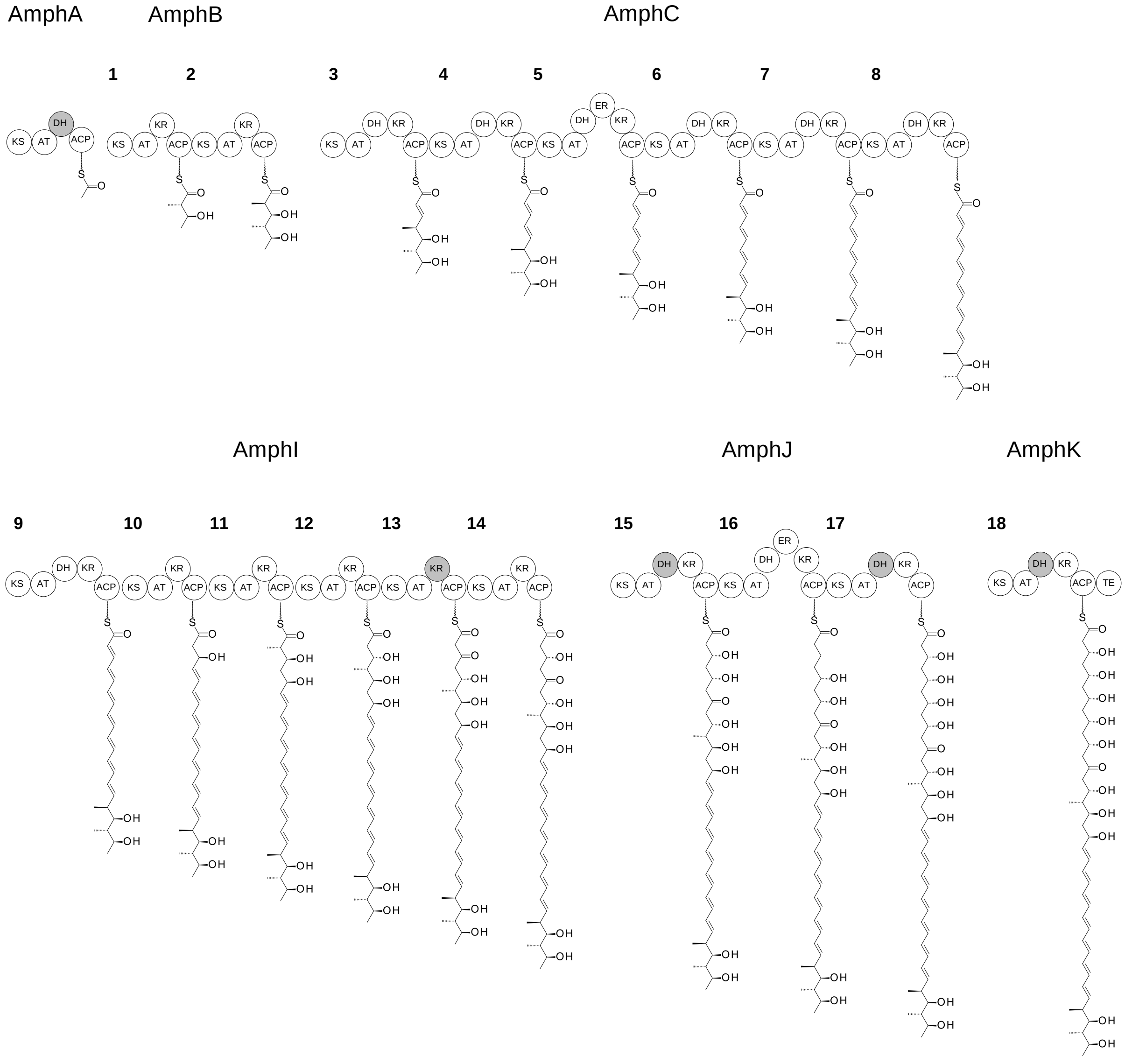

Fig. S1 The amphotericin PKS. The eighteen extension cycles shown give the polyketide that is cyclized to form the macrolactone of amphotericin B. The ER5 domain in AmphC module 5 is only partially active. As a result, the assembly line also produces the polyketide precursor of the tetraene amphotericin A, in which the C28-C29 alkene is reduced.

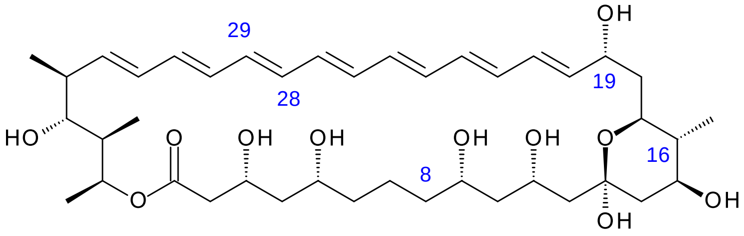

8-Deoxy-16-descarboxyl-16-methyl-amphoteronolide B

AmphN

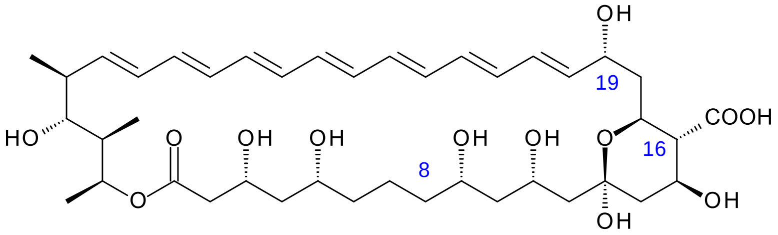

8-Deoxy-amphoteronolide B

AmphDI

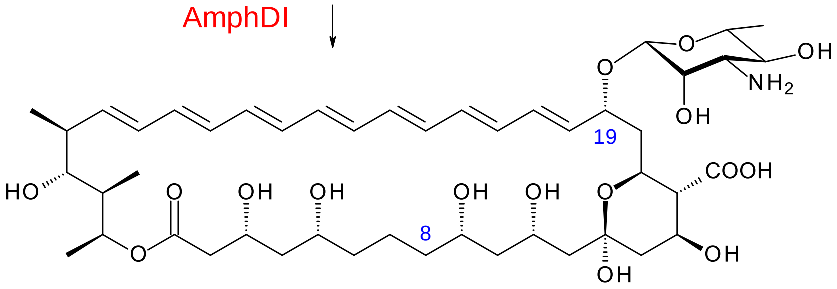

8-Deoxy-amphotericin B

AmphL

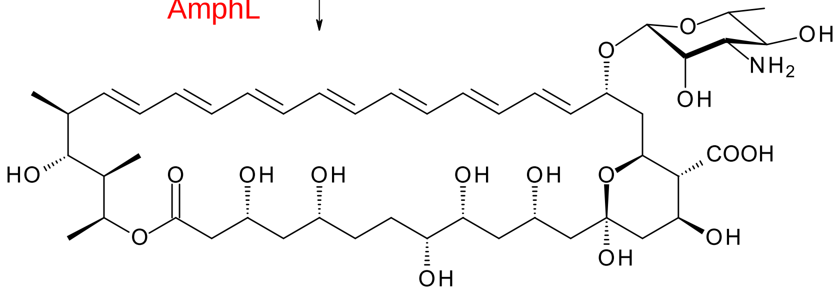

Amphotericin B

Fig. S2 Late stages in amphotericin biosynthesis. In the co-metabolite amphotericin A, the C28-C29 alkene is reduced.

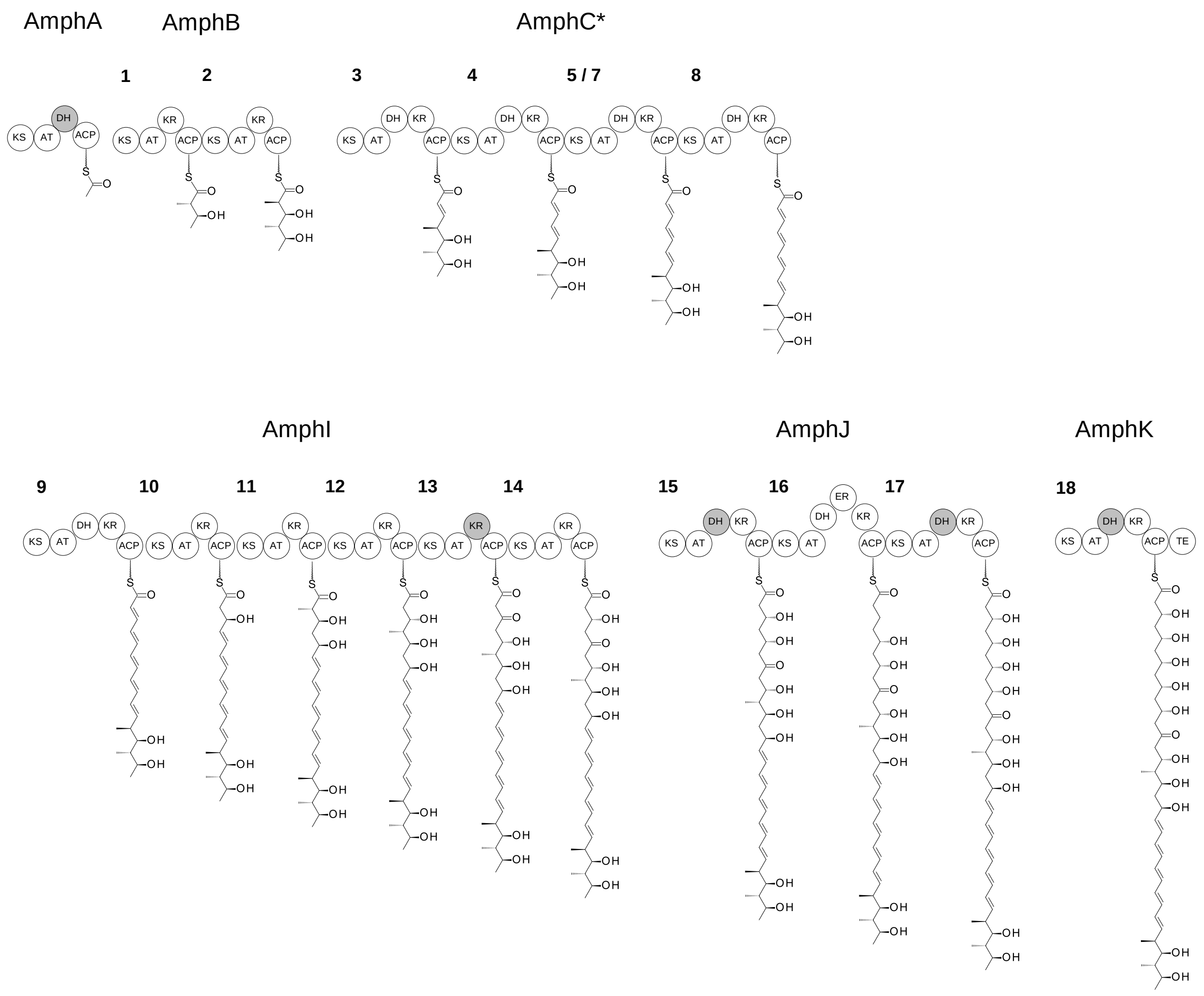

Fig. S3 *S. nodosus* M57 pentaene PKS. Modules 5, 6 and 7 are replaced with a hybrid 5/7 module [6].

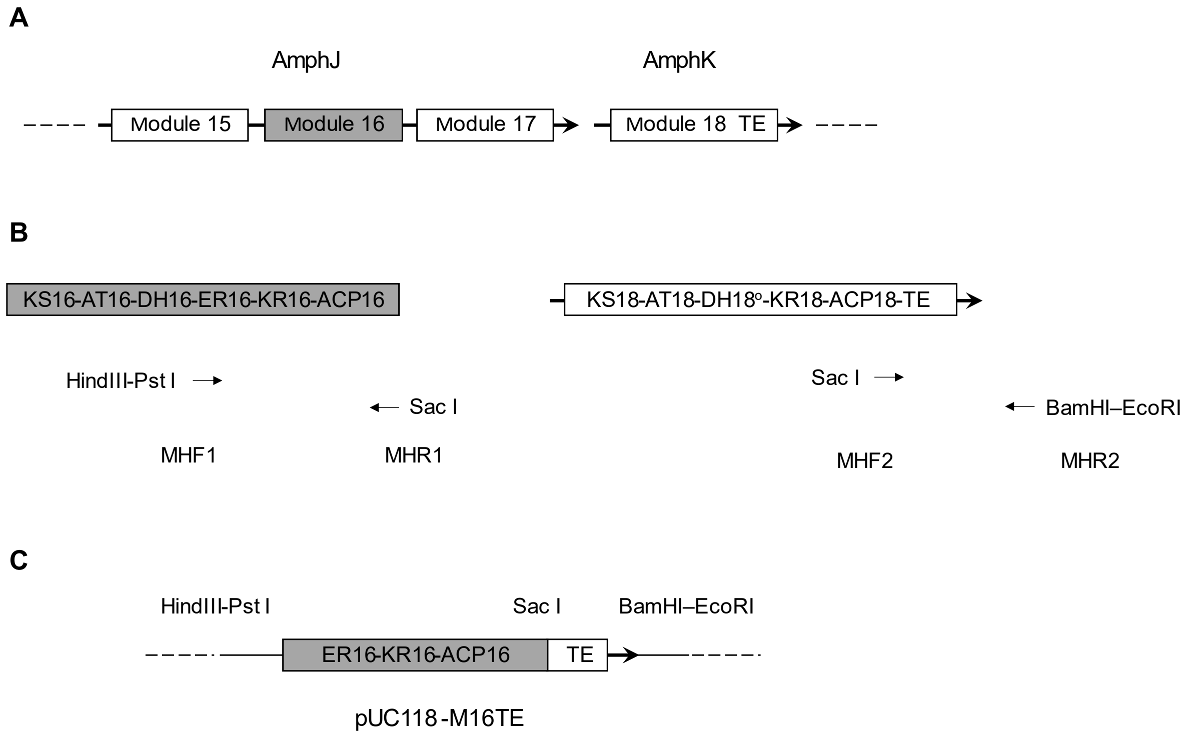

Fig. S4 Strategy for fusion of the coding sequences for ER16-KR16-ACP16 and the chain-terminating TE. (A) Schematic diagram of AmphJ and AmphK genes encoding modules 15 to 17 and module 18-TE, respectively. (B) Diagram showing annealing sites for MHF1 and MHR1 primers, used to amplify ER16-KR16-ACP16 coding sequence, and MHF2 and MHR2 primers, used to amplify the TE coding sequence. (C) Diagram showing pUC118-M16TE construct, which contains the DNA fragment encoding ER16-KR16-ACP16-TE.

A

```
ACP16   EFRGLGFDSLTAIELRNRLGKASGLTLTATLVFDYPTPQQLAEHLLDELLGADAAEAFAAPQTAA
ACP18   GFLELGFDSLVSVSLRNQLGELLGLRLPTSVVFDSKTPVKLARHLNEELGDLSASGPASGTAVAG--Thioesterase


Amphotericin ACP16-TE fusion
        EFRGLGFDSLTAIELRNRLGKASGLTLTATLVFDYPTPQQLAEHLLDELGDLSASGPASGTAVAG--Thioesterase

Stambomycin ACP13-TE fusion
        GFLELGMDSLTGVELRNRLAASTGLRLPATLVFDHPNCTELARRLAATTAADGQSPGSAVPAVTL--Thioesterase
```

B

```
ACP16

     D  Y  P  T  P  Q  Q  L  A  E  H  L  L  D  E  L  L  G  A  D
5’   GACTACCCGACGCCCCAGCAGCTCGCCGAGCACCTCCTGGACGAACTGCTCGGCGCCGAC 3’
                       GTCGAGCGGCTCGTGGAGGACCTGCTcGAgtctaga < MHR1



ACP18

    P  V  K  L  A  R  H  L  N  E  E  L  G  D  L  S  A  S  G  P
5’ CCGGTGAAGCTGGCCCGCCACCTCAACGAGGAACTGGGCGACCTGTCCGCCTCCGGCCCC 3’
                    MHF2 5’ AACGAGGAgCTcGGCGACCTGTCCGCCTCCGGCC 3’
```

Fig. S5 Fusion point between ACP16 and the chain-terminating TE in *S. nodosus* M57-16TE. A. Alignment of C-terminal regions of ACP16 and ACP18 domains. The phosphopantetheinylated serine residues are in bold text and the cross-over point is coloured red. The ACP16-TE fusion point is within 2 amino acid residues of the fusion point used in a productive ACP13-TE hybrid derived from the stambomycin PKS (Su et al., 2022). B. Sequences of MHR1 and MHF2 oligonucleotides aligned with the ACP coding sequences. The primers include a Sac I restriction that allows precise joining of the two PCR products.

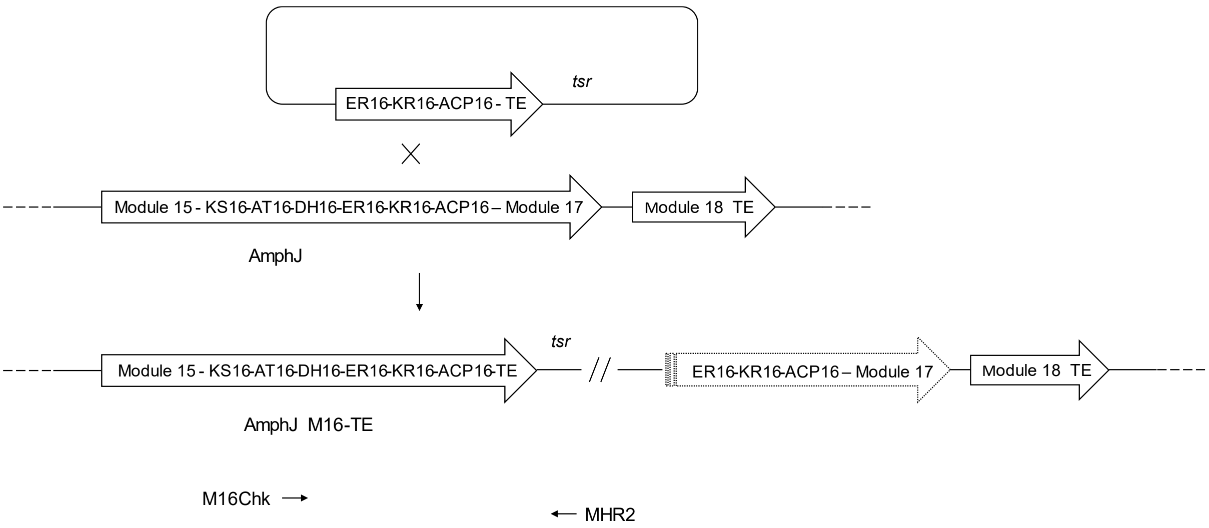

Fig. S6 Integration of KC-M16TE into the *S. nodosus* chromosome. Recombination between the vector-borne and chromosomal ER16-KR16-ACP-16 sequences duplicates this sequence within the genome of the recombinant. This integration event can be detected by PCR with M16 Chk, which anneals upstream from ER16, and primer MHR2, which anneals just after TE. These primers should amplify a 2984 bp product. Recombinants of this type should produce the target polyene with a contracted ring size.

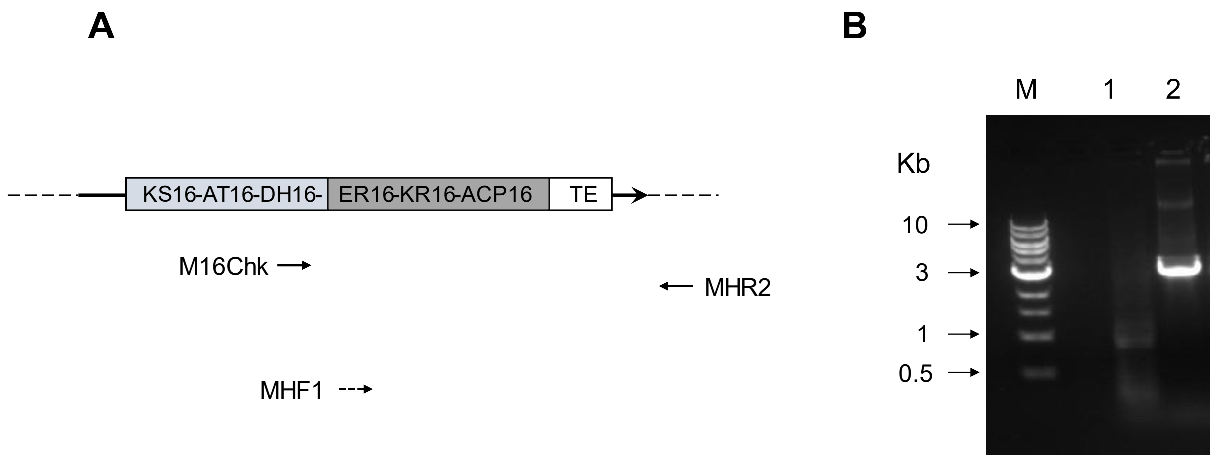

Fig. S7 PCR analysis of *S. nodosus* M57-16TE. PCRs were carried out with genomic DNA templates and primers M16Chk and MHR2 to identify recombinants resulting from cross-over between ER16-KR16-ACP16 sequences. The required clones give a strong band of 2984 bp. Lanes: M, molecular weight markers; 1, *S. nodosus* M57 genomic DNA (negative control); 2, *S. nodosus* M57-16TE.

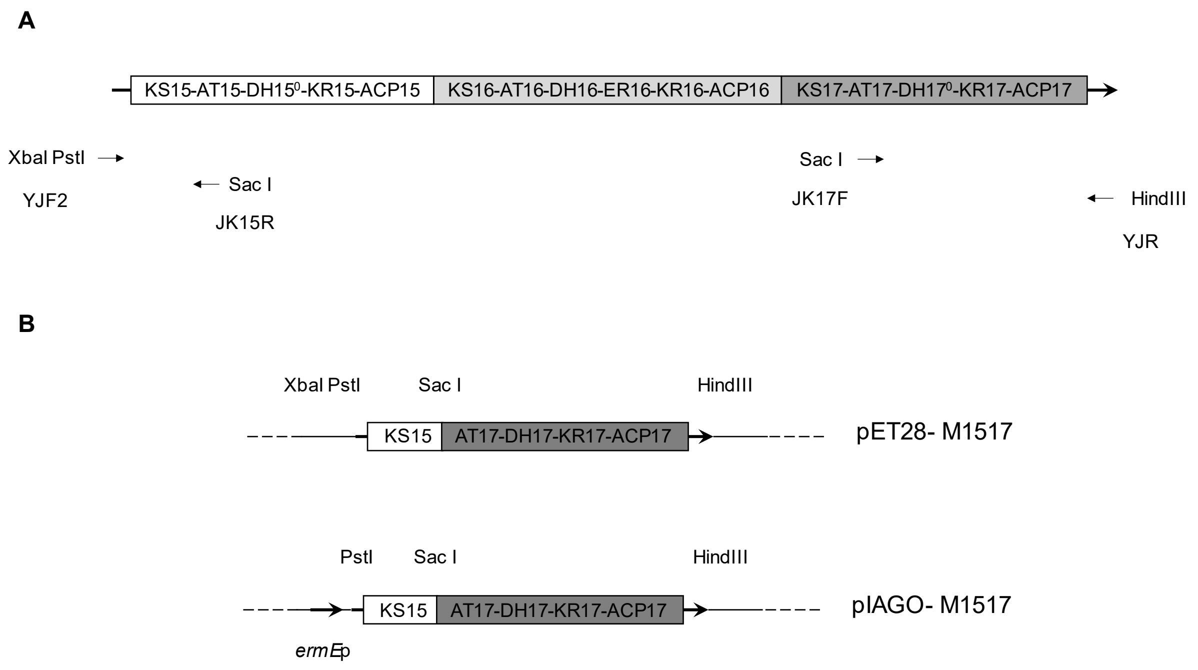

Fig. S8 Construction of a coding sequence for a single-module AmphJ* protein.

JK15R primer binding region

```
    R  P  R  R  S  G  V  S  S  F  G  V  S  G  T  N  A  H  V  I  V  E
5’ CGCCCCCGCCGCTCCGGCGTCTCCTCCTTCGGCGTCAGCGGCACCAACGCCCATGTCATCGTCGAG 3’
3’ GCGGGGGCGGCGAGGCCGCActcGAGGAAG  5’ < JK15R primer
```

JK17F primer binding region

```
   R  V  R  R  A  G  V  S  S  F  G  I  S  G  T  N  A  H  V  I  L  E
5’CGGGTCCGCAGGGCCGGTGTGTCCTCCTTCGGCATCAGCGGCACCAACGCCCATGTCATCCTCGAG 3’
JK17F primer > 5’GGTGTGagCTCCTTCGGCATCAGCGGCACCAAC 3’
```

KS15-KS17 junction

```
    R  P  R  R  S  G  V  S  S  F  G  I  S  G  T  N  A  H  V  I  L  E
5’ CGCCCCCGCCGCTCCGGCGTgagCTCCTTCGGCATCAGCGGCACCAACGCCCATGTCATCCTCGAG 3’
```

Fig. S9 Fusion point between KS15 and AT17 in *S. nodosus* M57-1517.

A

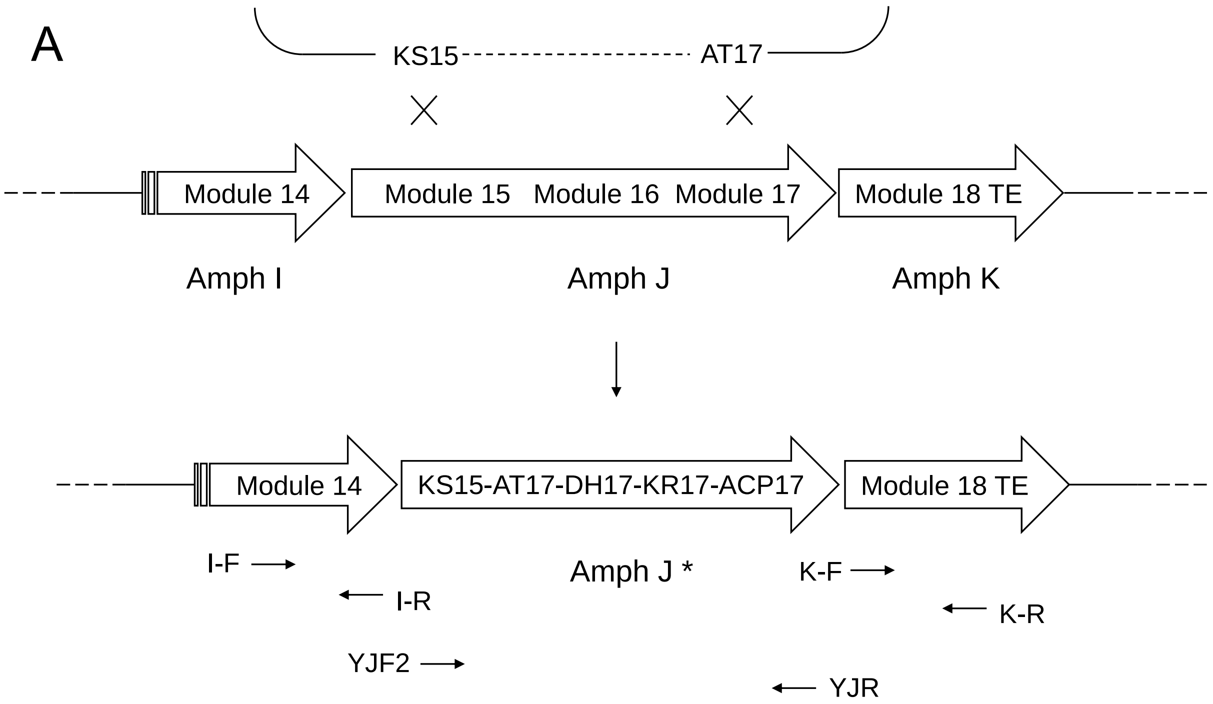

B

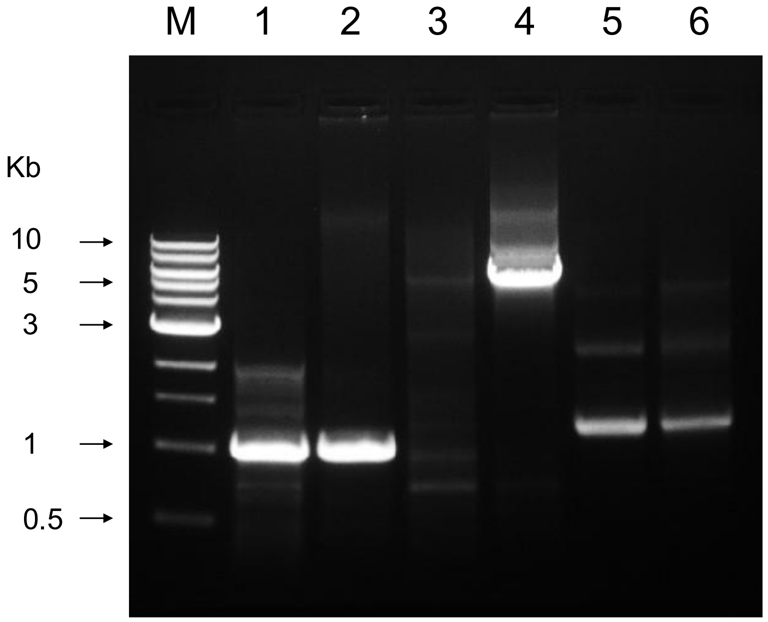

Fig. S10 Replacement of gene for trimodular AmphJ protein with gene for single-module AmphJ* protein. A. Schematic diagram showing how two recombination events lead to gene replacement. B. PCR analysis of chromosomal DNA from *S. nodosus* M57-1517. PCR with I-F and I-R primers reveals that the 3′ end of the *amphI* gene is present in M57 and M57-1517 (lanes 1 and 2). PCR with primers YJF2 and YJR reveal that a 5kb gene for AmphJ* is present in M57-1517 (lane 4) but not M57 (lane 3). These primers do not amplify the 15kb *amphJ* gene from M57. PCR with K-F and K-R primers revealed that the 5′ end of the *amphK* gene is present in both M57 and M57-1517 (lanes 5 and 6).

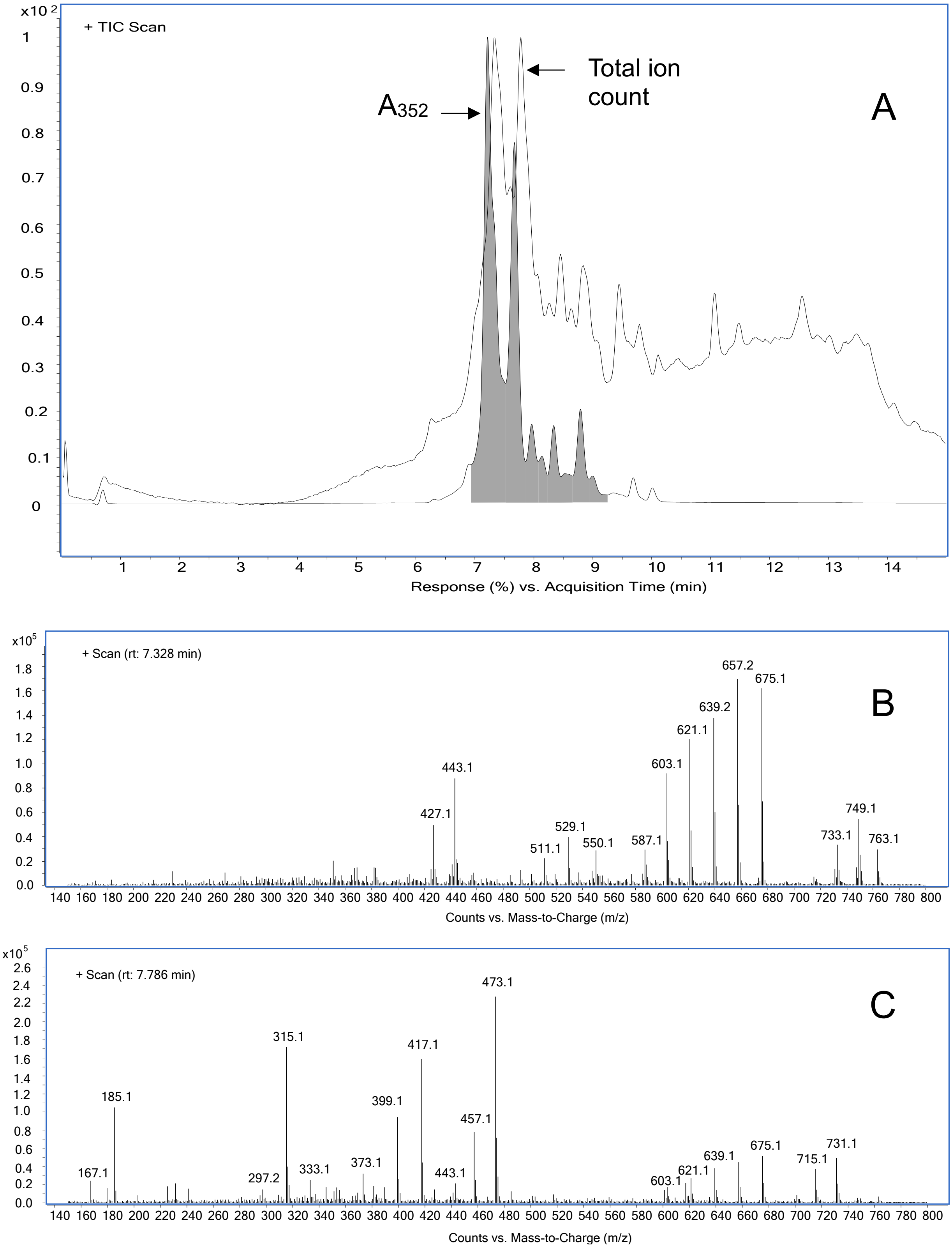

Fig. S11 LCMS analysis of pentaenes from *S. nodosus* M57 M16-TE. A. LC chromatogram, showing total ion count and A_{352} versus time. The main pentaene (A_{352}) peaks are shaded grey. B. Ion spectrum of leading pentaene peak, RT = 7.328 min. C. Ion spectrum of second pentaene peak, RT = 7.786 min.

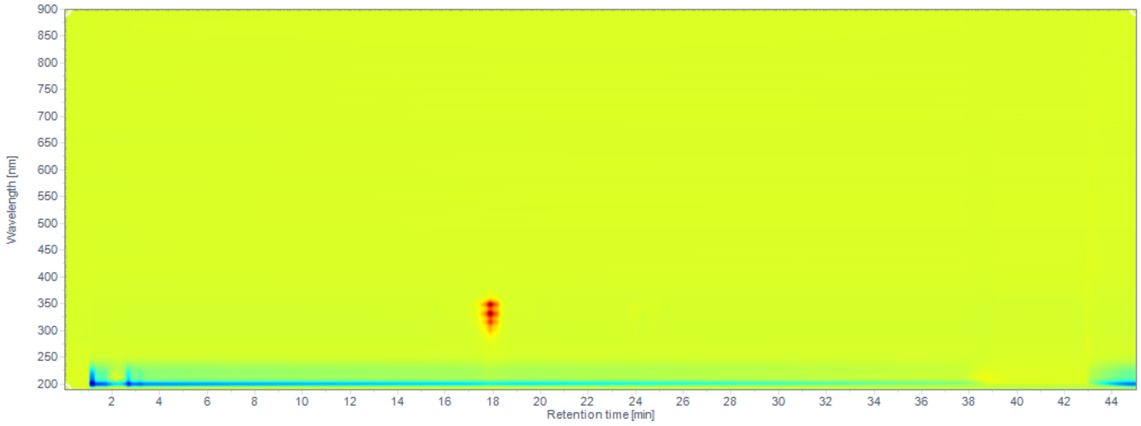

Fig. S12. HPLC analysis of highly purified pentaene from M57-1517. The contour plot of the diode array display shows the pentaene peak at 18 min. No other compounds that absorb in the UV-visible region were detected.

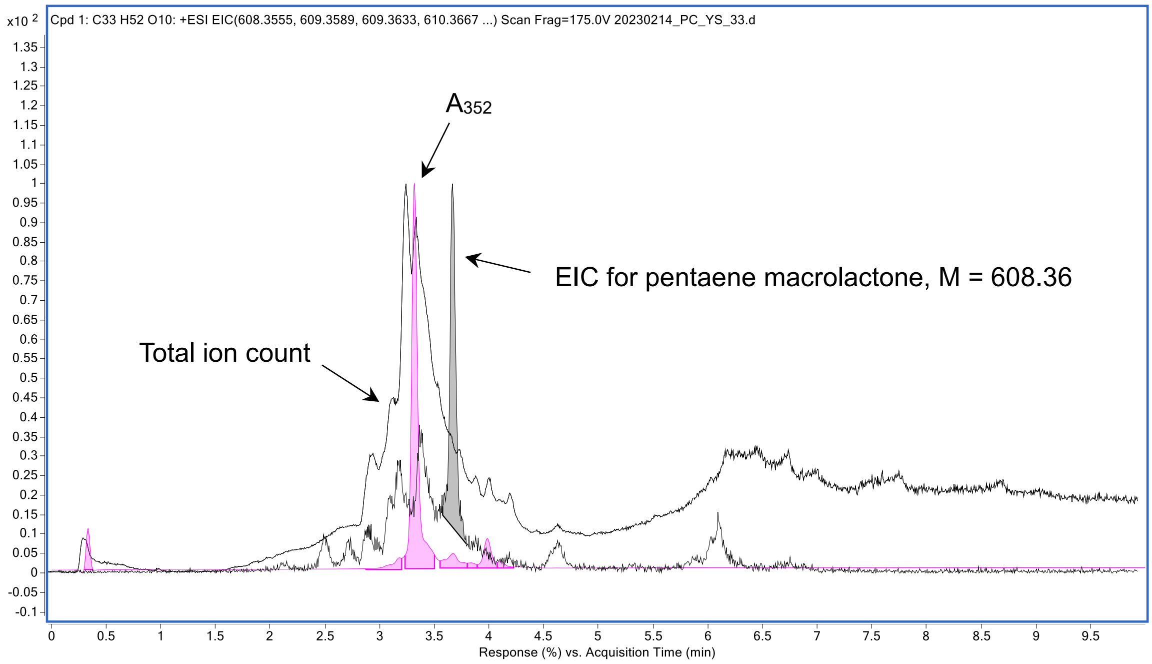

Fig. S13 LC-MS analysis of HPLC-purified pentaene. The chromatogram shows total ion count, total pentaene A_{352} (magenta), and the extracted ion chromatogram for $C_{33}H_{52}O_{10}$, the molecular formula of the pentaene macrolactone predicted as the full-length cyclised product of the engineered PKS in strain M57-1517. The major peak in this EIC coincides with a minor pentaene A_{352} peak.

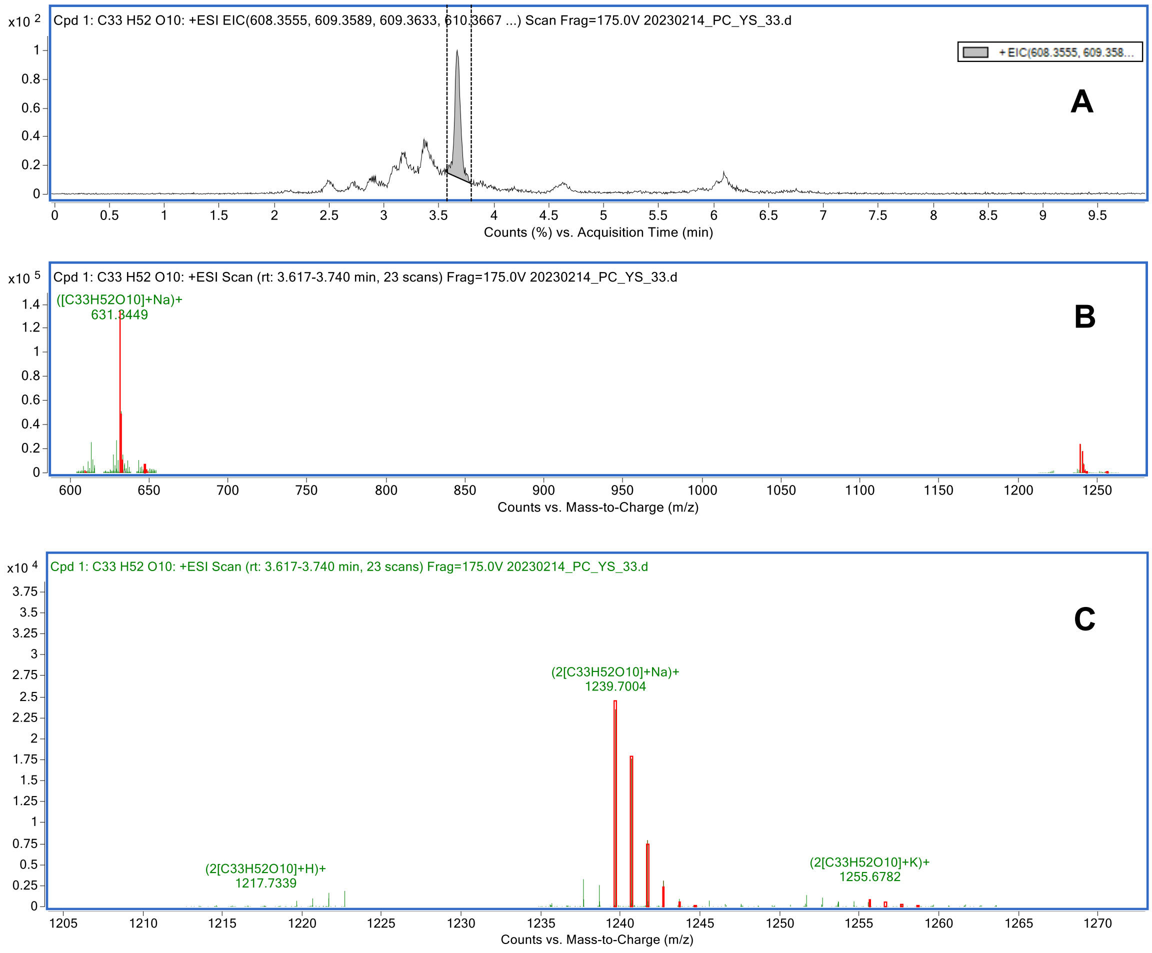

Fig. S14 Detection of pentaene macrolactone predicted as the full-length cyclised product of the engineered PKS. A. Extracted ion chromatogram for $C_{33}H_{52}O_{10}$, the molecular formula of the full-length cyclised pentaene macrolactone. B. Ion spectrum of the major peak in panel A in the m/z range 600 to 1250. The sodium adduct of the full-length pentaene macrolactone is present, $(C_{33}H_{52}O_{10} + Na)^+$ = 631.3449. C. Expansion of the ion spectrum in panel B in the range 1205 to 1270. A singly charged dimer of the full-length pentaene macrolactone is present as a sodium adduct, $(2[C_{33}H_{52}O_{10}] + Na)^+$ = 1239.7004.

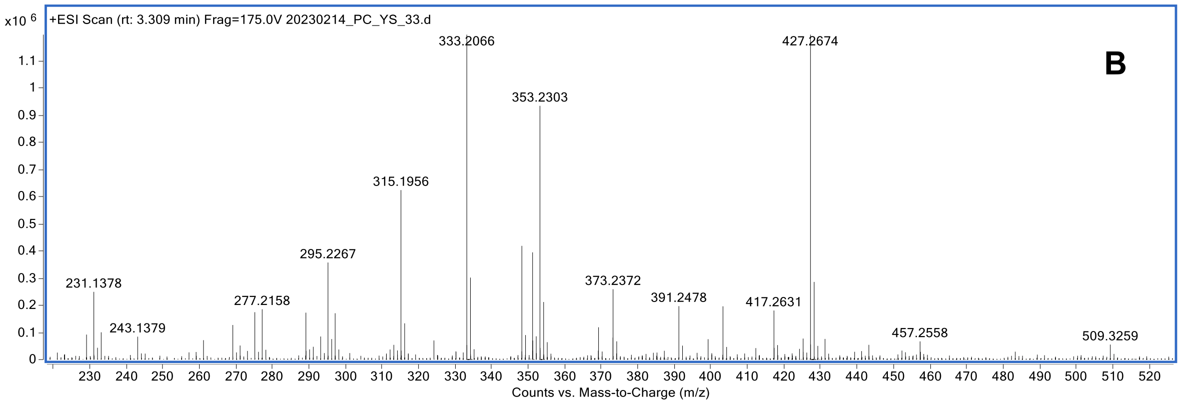

Fig. S15 Positive ion mass spectrum of the major A_{352} pentaene peak from *S. nodosus* M57-517.

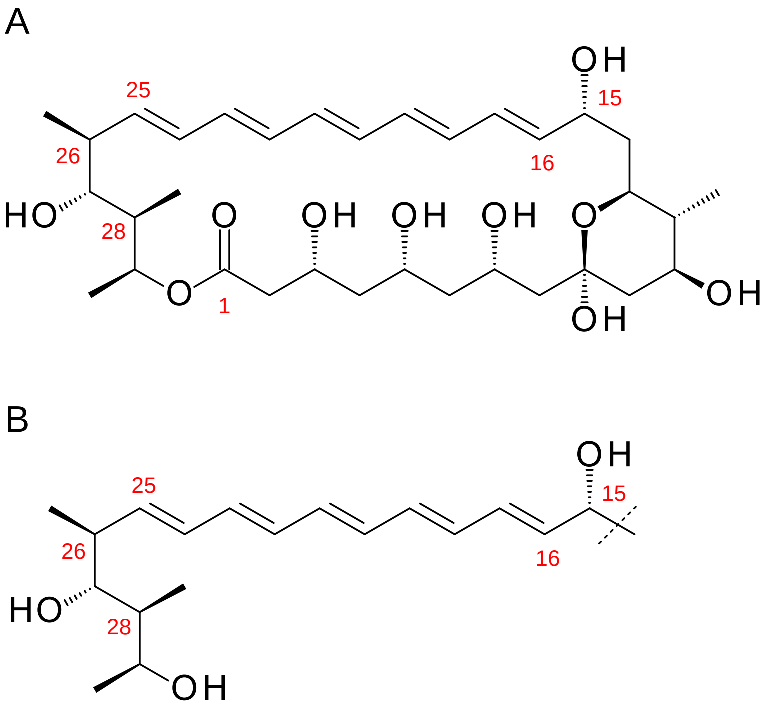

Fig. S16 Ring numbering for full-length pentaene macrolactone from *S. nodosus* M57-1517. B. Fragment common to biosynthetic intermediates detected in the purified pentaene sample by HR-MS. This numbering system is used in analysing the NMR data.

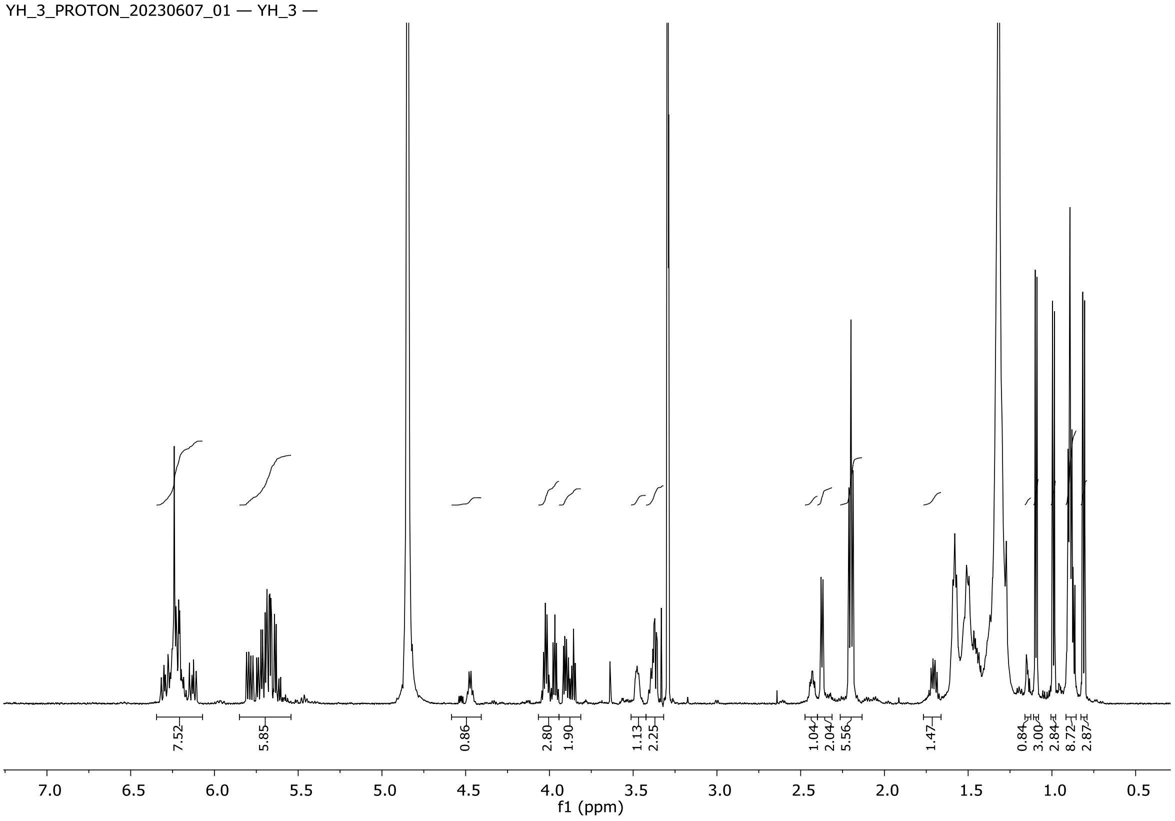

Fig. S17. Proton NMR spectrum of highly purified pentaene from *S. nodosus* M57-1517. The spectrum shows features characteristic of a polyene macrolactone, such as the peaks between 6.5 and 5.5 ppm. With polyene macrolides in general, signals in this region result from alkenyl protons attached to the series of conjugated double bonds. The pentaene contains methyl groups derived from the acetyl primer and from methylmalonyl CoA-derived propionate extender units. The three protons of a given methyl branch absorb in the region between 0.8 and 1.2 ppm. For each methyl group (or set of equivalent methyl protons) the peak is split into a doublet by the single proton attached to the adjacent methine carbon atom. The total peak area of a methyl doublet corresponds to three protons and can be used to determine the numbers of protons contributing to other pentaene signals.

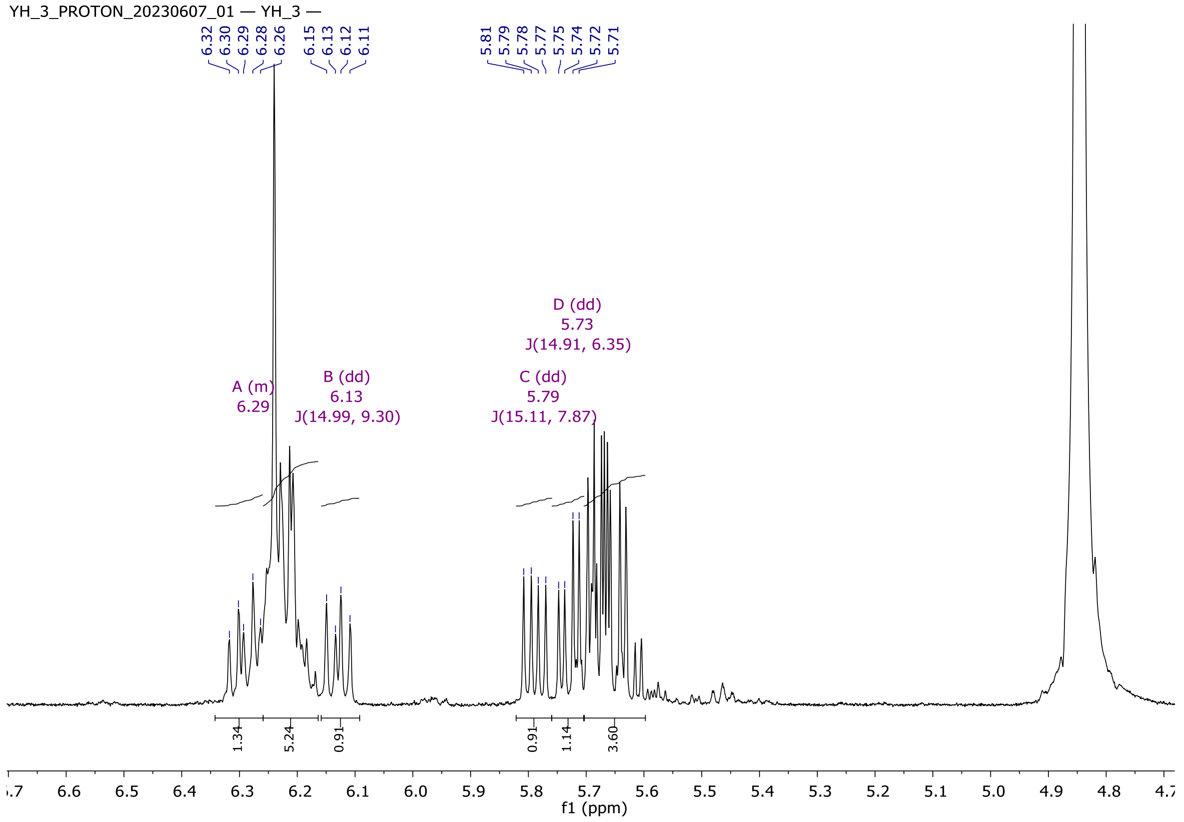

Fig. S18 Expansion of the 6.6 to 4.7 ppm region of the proton NMR spectrum of the purified pentaene. The large signal at 4.85 ppm derives from water in the NMR solvent. There are two multiplets between 6.35 and 6.10 and between 5.85 and 5.60 ppm, the region where alkenyl protons absorb. Each of these multiplets derives from 5 protons as determined by comparing peak areas with those of the methyl doublet at 1.10 ppm.

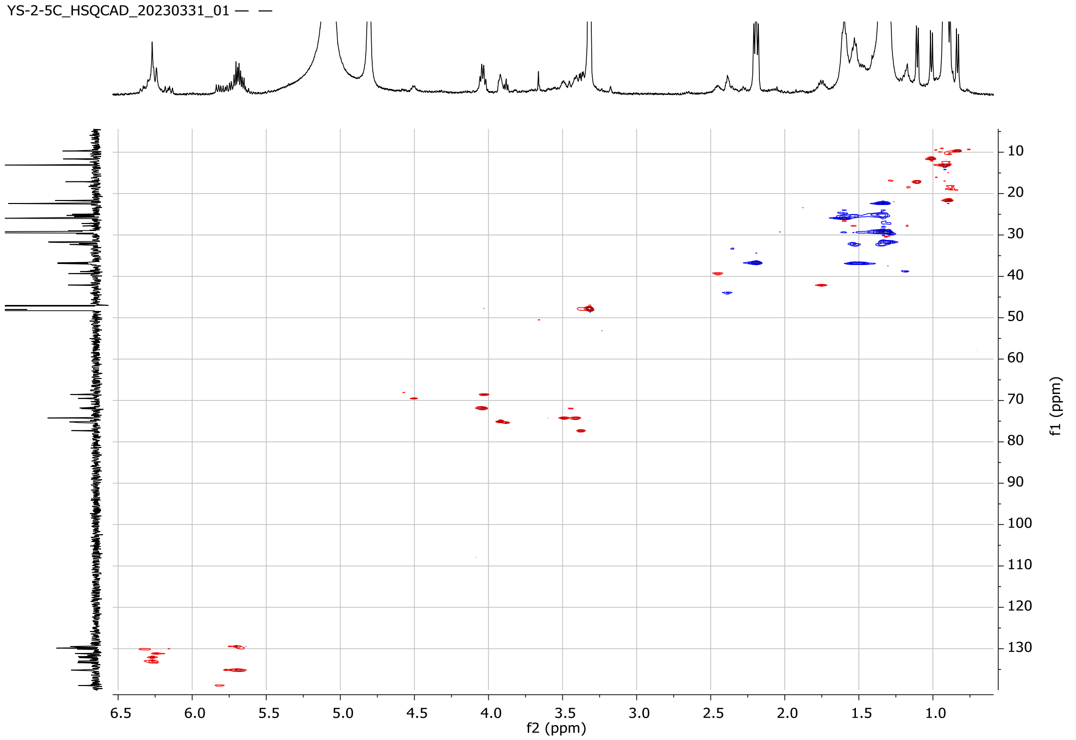

Fig. S19 ^{1}H-^{13}C-HSQC NMR spectrum (0.2-6.5 ppm/10-140 ppm region) for pentaene (500 MHz, CD_3OD, 25 °C). Heteronuclear single quantum coherence (HSQC) spectroscopy determines proton-carbon single bond correlations. Alkenyl protons giving the 6.4 to 6.1 signals correlate with 5 carbon atoms resonating between 130 to 133.5 ppm. These ^{13}C chemical shifts are consistent with alkenyl carbon atoms. The complex multiplet between 5.85 and 5.60 ppm contains peaks that correlate with 4 to 5 carbon signals (140 to 130 ppm). Together this evidence supports the assignment of the conjugated pentaene in the putative structure, i. e. possessing 10 protons and 10 carbons all in different chemical environments (red correlations indicate CH or CH_3 units, whereas blue correlations represent CH_2 groups).

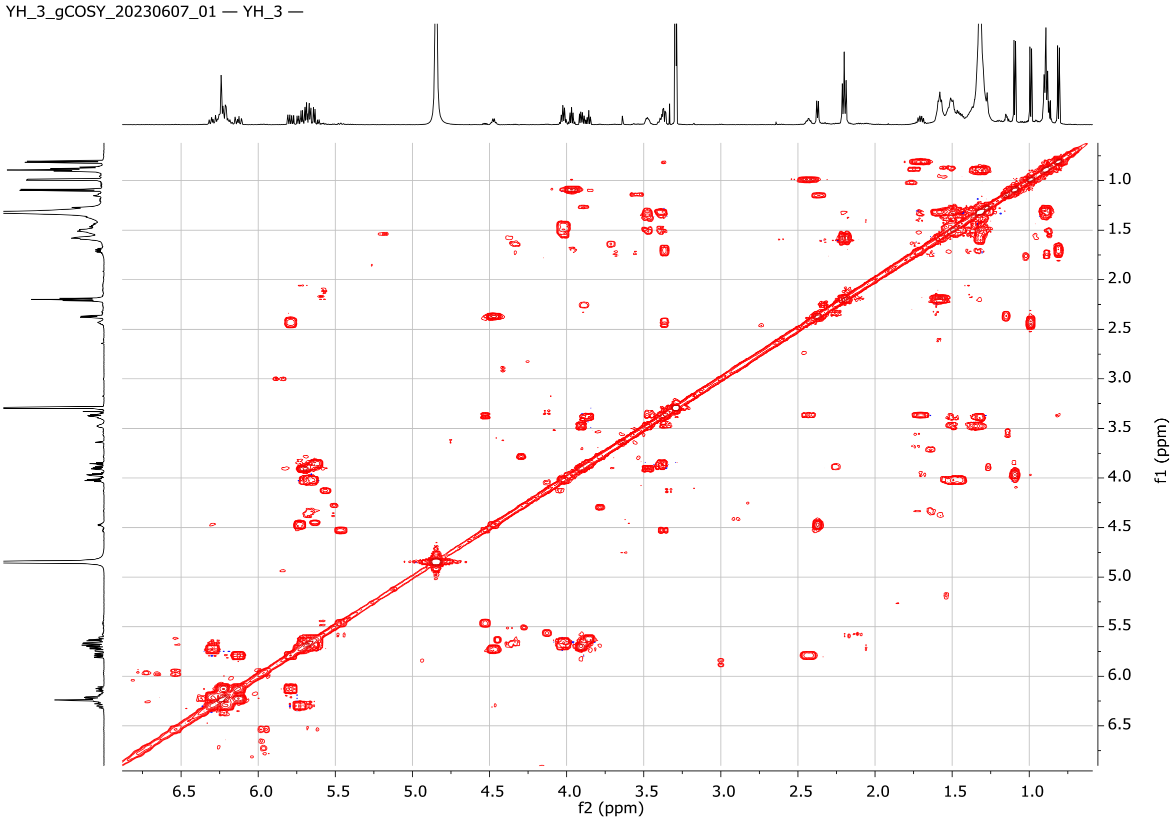

Fig. S20 ^1H-^1H-gCOSY NMR spectrum for the purified pentaene (500 MHz, CD_3OD, 25 °C). H1-H1 correlation spectroscopy (COSY) detects interactions between specific hydrogen atoms. There are the expected correlations between signals in the 6.35-6.10 ppm region and those between 5.85-5.60 ppm. The downfield multiplet (6.35-6.10 ppm) does not correlate with any other signals. However, the multiplet at 5.85-5.60 ppm correlates to several upfield signals which would be consistent with protons at either end of the polyene, i.e., C-25 and C-16. For example, a contribution at 5.80 ppm correlates to a signal at 2.50 ppm assigned to the allylic proton at C-26 (see below) and is indicative of a C-25 to C-26 connection. A contribution from 5.70 ppm correlates to signals between 4.05 ppm and 3.80 ppm. This supports a C-16 to C-15 connection, C-15 being a methine attached to oxygen.

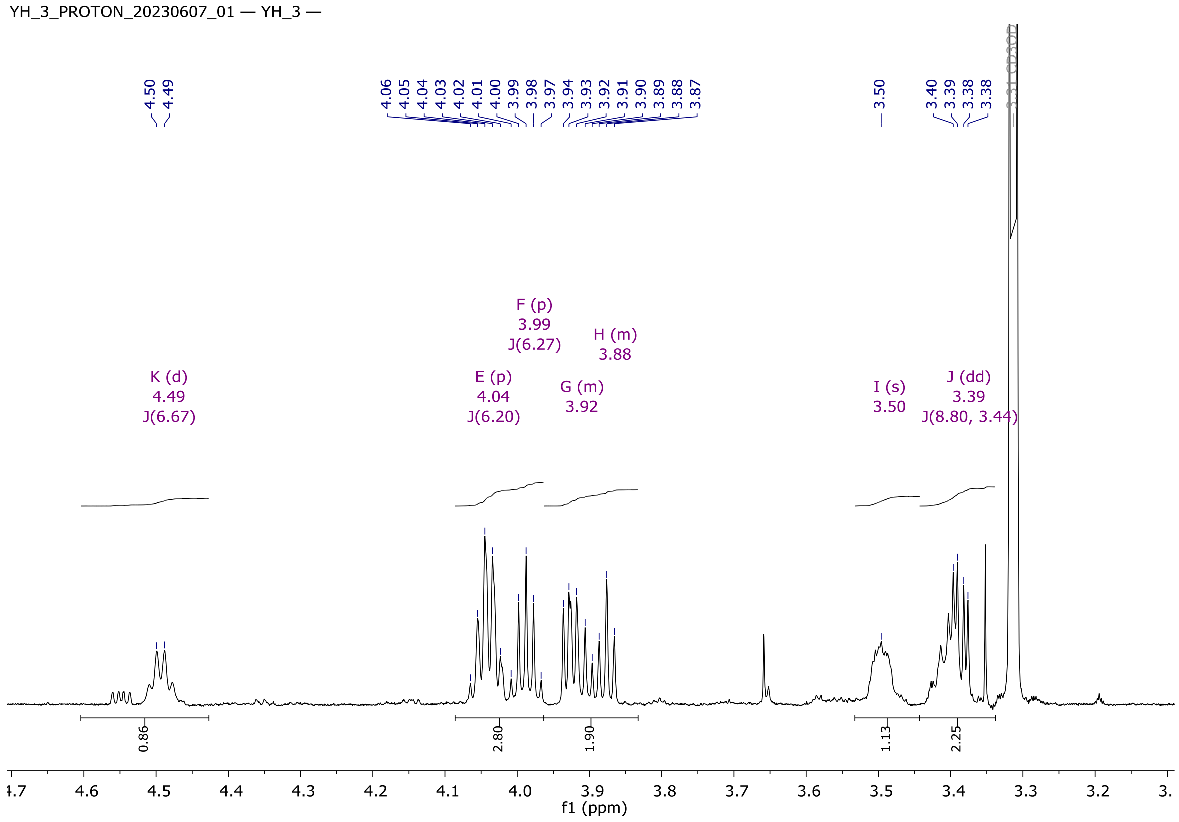

Fig. S21 Expansion of the 4.7 to 3.1 ppm region of the proton NMR spectrum of the purified pentaene. This region includes the signal for residual protonated CD_3OD (i. e. CD_2HOD) at 3.30 ppm. Apart from this there are several distinct signals. At 4.05 ppm there is an apparent quartet that integrates to 2.5 protons. In the HSQC spectrum (Fig. S19) this correlates to two distinct CH carbons at approx. 70 ppm. Based on the chemical shifts of both the protons and carbons, it can be concluded that these are consistent with methines which bear an oxygen atom (i. e. hydroxy groups). In the ^{1}H-^{1}H-COSY this 4.05 ppm signal correlates with a methyl group at 1.10 ppm (Fig. S16). These data are consistent with an assignment of one of the protons as H-29 and the carbon as C-29. The other correlations from 4.05 ppm are to an alkenyl proton at 5.60 ppm and with a signal at 1.50 ppm. This may be H-15.

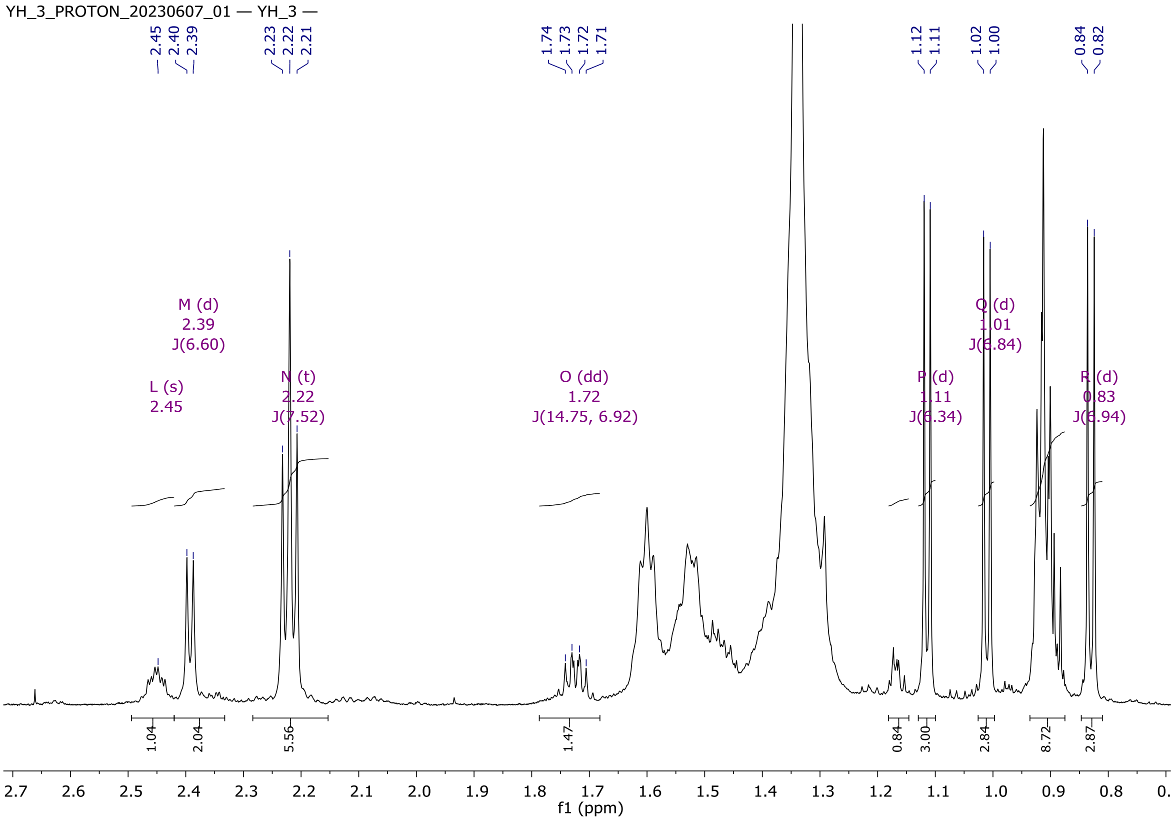

Fig. S22 Expansion of the 2.7 to 0.8 ppm region of the proton NMR spectrum of the purified pentaene. At 2.45 ppm there is a broad signal which integrates to 1 proton and correlates to 1 CH at approx. 38 ppm in the HSQC spectrum (Fig. S15). In the COSY spectrum (Fig. S16) it couples to one of the alkenyl protons and also to the methyl doublet at 1.00 ppm. This allows confident assignment of these signals to the allylic position C-26. The next signal at 2.40 ppm is likely due to an impurity. The triplet at 2.20 ppm is typical of a methylene group (CH_2) adjacent to a carbonyl group in a compound such as a fatty acid, a non-UV active impurity in this sample.

At 1.75 ppm there is a broad signal which integrates to 1 proton and correlates to one CH at 42.5 ppm in the HSQC. In the COSY spectrum (Fig. S16) this clearly correlates to the methyl doublet at 0.83 ppm and is consistent with a propionate methyl/methine e. g. C-28.

Between 1.60 ppm and 1.20 ppm there is a large signal which may include some signals of interest from the putative polyketide but is obscured by the methylene groups in the fatty acid impurities. In the region 1.10 ppm to 0.8 ppm there are three clear doublets for 3 methyl groups in the polyketide. These correlate in the COSY to three distinct methines as mentioned above. They also clearly correlate to 3 methyl signals in the HSQC at 17.5 ppm, 12 ppm and 10 ppm respectively. The putative structure contains four, not three, methyl groups. There is no clear evidence for a fourth methyl group. In relation to this there is a large and broad signal between 0.95 ppm and 0.85 ppm. Expansion of the proton NMR spectrum suggests that more than one methyl group has produced this signal. In the HSQC it looks like this signal correlates with signals at 13 ppm and 22.5 ppm and at least in part this is caused by the terminal methyl groups in the fatty acid impurities.

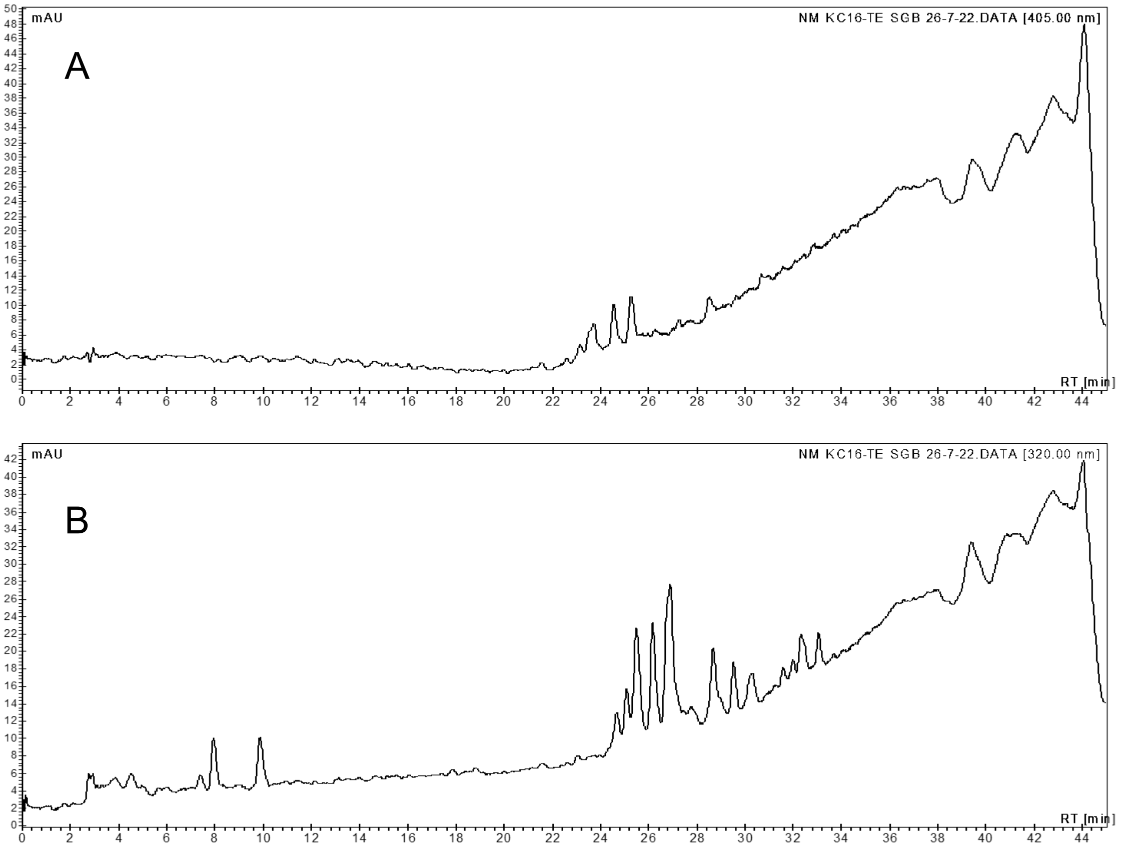

Fig. S23 HPLC analysis of polyenes from *S. nodosus* NM-16TE. A, heptaene; B, tetraene. Very low levels of these polyenes were detected with heptaene peaks giving only about 12 milli-absorbance units (mAU) and tetraene peaks giving only about 28 milli-absorbance units (mAU).

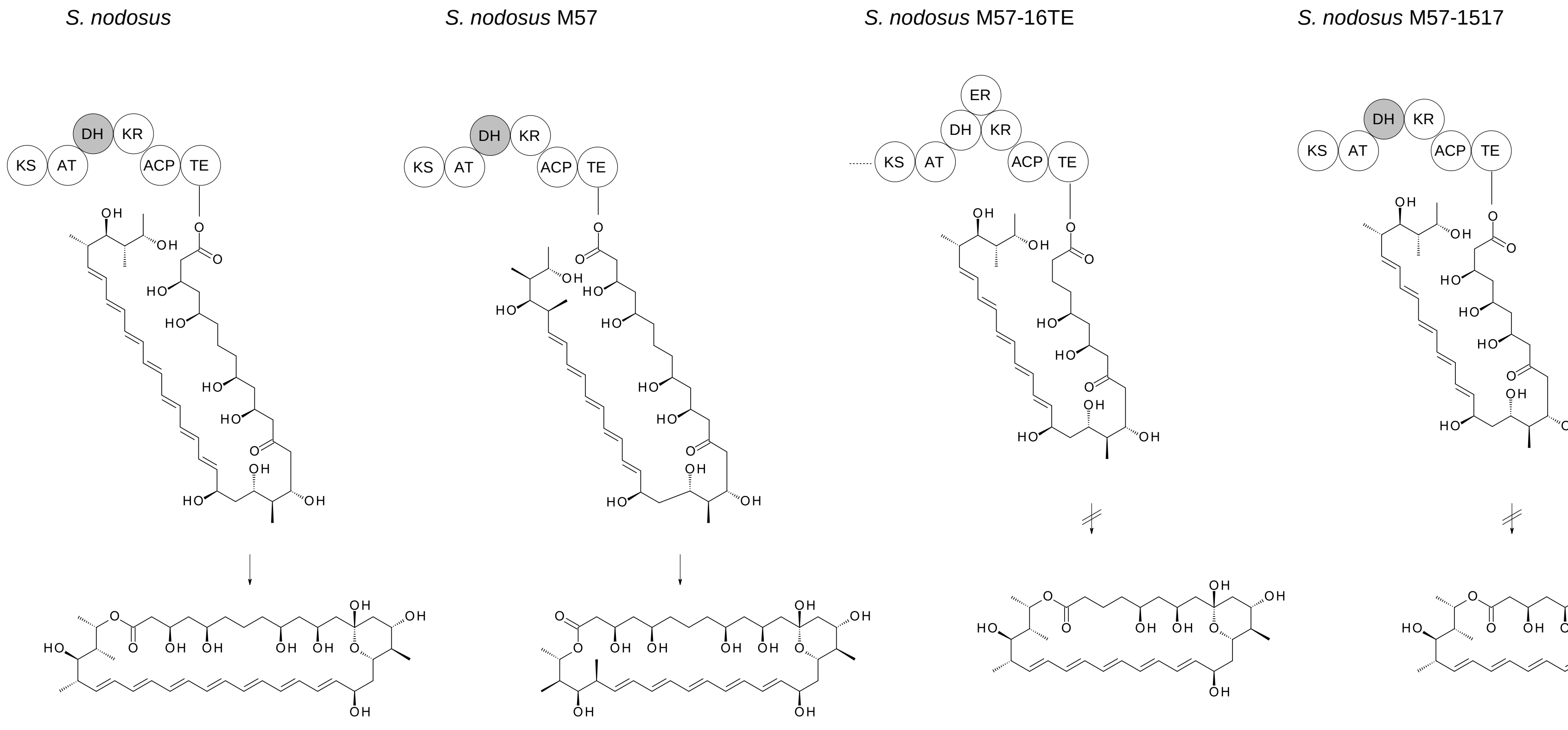

Fig. S24 Schematic diagram showing action of amphotericin PKS TE against various substrates.
